# Tissue-Agnostic Targeting in Solid Tumors: A PRISMA-Compliant Meta-Analysis of Efficacy, Safety, and Resistance Determinants Across Histologies

**DOI:** 10.32604/or.2026.077965

**Published:** 2026-06-16

**Authors:** Marwa Balaha, Saad A. Aldosari, Ahmed A. Alamer, Nehad Ahmed, Mohamed F. Balaha

**Affiliations:** 1 Department of Pharmacy, “G. d’Annunzio” University of Chieti-Pescara, Chieti, Italy; 2 Department of Clinical Pharmacy, College of Pharmacy, Prince Sattam Bin Abdulaziz University, Al-Kharj, Saudi Arabia

**Keywords:** Tissue-agnostic therapy, PRISMA, meta-analysis, MSI-H/dMMR, PD-1 inhibitor, HER2-low, antibody-drug conjugate, interstitial lung disease, BRAF V600E, NTRK fusion

## Abstract

**Objectives:** Tissue-agnostic oncology personalizes treatments based on shared molecular biomarkers, addressing challenges like assay variability, control-arm rigor, and non-proportional hazards. Integrating efficacy, safety, and resistance factors with consistent estimands is essential for evaluating biomarker-matched therapies across histologies. This review aims to quantify and compare their efficacy and safety, and to identify determinants of resistance, using PRISMA-compliant methods. **Methods:** We conducted a systematic review and random-effects meta-analysis of 38 studies (15,018 participants), employing dual screening, standardized bias assessment, and evaluations of heterogeneity and small-study effects. Hazard ratios (HRs) with 95% CIs were estimated for time-to-event outcomes, and restricted mean survival times were used when the proportional hazards assumption was violated. **Results:** Trastuzumab deruxtecan improved objective response rates and extended progression-free survival (PFS) and overall survival (OS) in HER2-positive gastric and gastroesophageal junction cancers and in HER2-low metastatic breast cancer, showing longer response durations. In metastatic colorectal cancer with microsatellite instability-high (MSI-H) or deficient mismatch repair (dMMR), PD-1 blockade significantly increased PFS and five-year OS despite crossover, with restricted mean survival time gains of about 11 months. In endometrial cancer, dostarlimab combined with chemotherapy improved PFS in both dMMR/MSI-H and mismatch repair–proficient disease and increased OS overall. Encorafenib-based therapies reduced progression and death in BRAF V600E metastatic colorectal cancer. Safety profiles were class-specific: PD-1 inhibitors caused fewer grade 3 or higher adverse events than chemotherapy, whereas trastuzumab deruxtecan was associated with increased interstitial lung disease (ILD) or pneumonitis and higher rates of treatment discontinuation. **Conclusion:** Biomarker-matched therapies confer significant survival benefits with predictable toxicities. Confidence is strongest for PD-1 inhibitors in MSI-H/dMMR tumors, trastuzumab deruxtecan in HER2-low or HER2-positive cancers, and encorafenib-based regimens in BRAF V600E metastatic colorectal cancer. Implementation should include validated assays (including reconfirmation of HER2 status), prioritize earlier treatment lines where gains are greatest, and require vigilant ILD monitoring. Head-to-head trials and assay standardization, especially for tumor mutational burden, remain priorities.

## 1 Introduction

Precision oncology is increasingly guided by tissue-agnostic selection, aligning therapies with actionable biomarkers regardless of histology. Robust evidence supports this approach across microsatellite instability-high/deficient mismatch repair (MSI-H/dMMR), high tumor mutational burden (TMB), neurotrophic tyrosine receptor kinase (NTRK) fusions, BRAF V600 variants, and human epidermal growth factor receptor 2 (HER2)-positive/low expression. In MSI-H/dMMR metastatic colorectal cancer (mCRC), first-line programmed cell death-1 (PD-1) blockade improves progression-free survival (PFS) compared to chemotherapy and provides durable control; dual checkpoint inhibition further prolongs survival under non-proportional hazards [1,2]. TMB-high status prospectively enriches for immunotherapy benefit across various histologies, although predictive performance varies by platform and threshold [3]. NTRK1/2/3 fusions define a rare pan-tumor class with high response rates; indirect comparison data suggest differences in complete response and durability among first-generation TRK inhibitors [4]. HER2-targeted antibody-drug conjugates (ADCs) expand benefits from HER2-positive to HER2-low breast cancer and outperform active second-line regimens in HER2-positive gastric and gastroesophageal junction (GEJ) cancer [5,6,7]. In BRAF V600E metastatic colorectal cancer (mCRC), regimens combining epidermal growth factor receptor (EGFR) blockade with or without chemotherapy have improved PFS and overall survival (OS) across multiple lines of therapy [8,9]. In this review, tissue-agnostic is used operationally to denote biomarker-defined treatment strategies in which eligibility and clinical rationale are not inherently restricted to a single histology (e.g., MSI-H/dMMR, TMB-high, NTRK fusions in regulatory/clinical contexts). These are distinguished from biomarker-guided strategies that remain substantially tumor-type-anchored in their evidence base and use (e.g., HER2-targeted ADCs in breast and gastric/GEJ cancer; BRAF V600E regimens primarily evaluated in colorectal cancer).

Clinical implementation remains difficult due to variability in biomarker detection, comparator strength, and endpoint evaluation. MSI/dMMR tests include immunohistochemistry (IHC), polymerase chain reaction (PCR), and next-generation sequencing (NGS), each of which affects classification and trial eligibility; MSI-H prevalence in gastric/GEJ varies by treatment line and region [10]. In endometrial cancer, coexisting genomic features, such as polymerase proofreading defects, influence immune response [11]. Regulatory guidance and approvals for MSI-H/dMMR testing and first-line PD-1 therapy also shape clinical practices [12]. HER2 positivity should be confirmed again after trastuzumab in gastric/GEJ cancer; ADCs show a significant risk of interstitial lung disease (ILD), requiring monitoring [6]. This PRISMA-compliant meta-analysis combines data on effectiveness, safety, and resistance mechanisms across tumor types to guide assay-informed patient selection, early-line use where maximum benefit is observed, and the selection of specific monitoring strategies. The aim of this study was to systematically synthesize comparative evidence on the efficacy and safety of biomarker-matched therapies across eligible solid-tumor contexts and to summarize reported resistance determinants using PRISMA-compliant methods.

## 2 Methods

**Figure 1:**
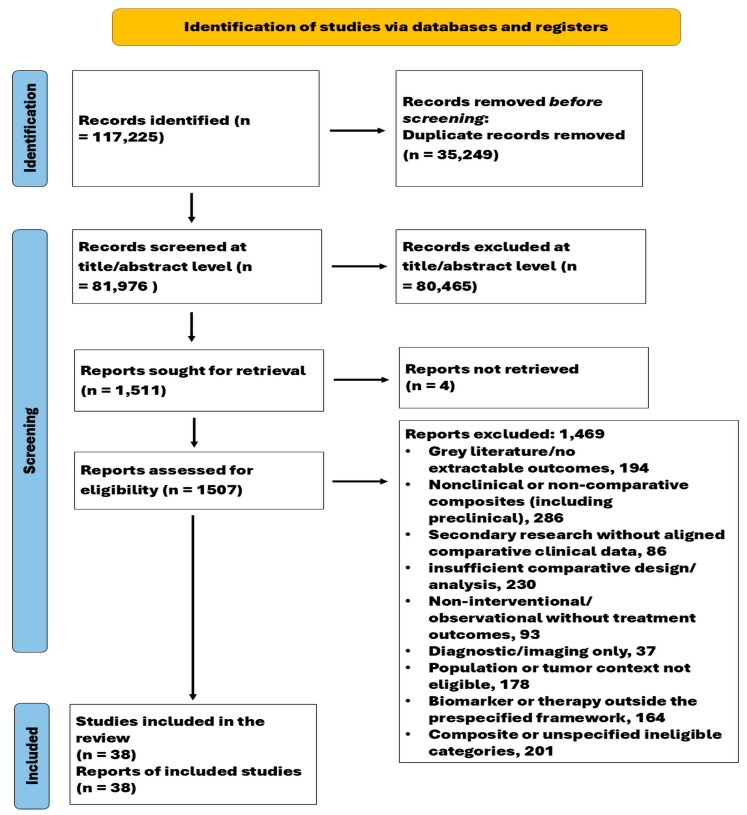
PRISMA-flow diagram of study selection: 117,225 records identified; 35,249 duplicates removed, leaving 81,976 records screened; 80,465 excluded. 1511 reports sought; 4 not retrieved; 1507 assessed; 1469 excluded for reasons including grey literature/no extractable outcomes (194); nonclinical or non-comparative composites (286); secondary research without aligned data (86); insufficient design/analysis (230); non-interventional/observational without outcomes (93); diagnostic/imaging only (37); unqualified population or tumor context (178); biomarker or therapy outside framework (164); composite/unspecified ineligible (201). 38 studies included.

This review adhered to Preferred Reporting Items for Systematic Reviews and Meta-Analyses (PRISMA) 2020 and PRISMA-S guidelines. A completed PRISMA checklist and PRISMA flow diagram are included in the Supplementary Materials and Fig. 1. Since this meta-analysis combined published, de-identified data in aggregate, institutional review board approval and informed consent were not necessary.

### 2.1 Protocol and Registration

The study followed the PRISMA 2020 guidelines and was prospectively registered on PROSPERO (CRD420251235402). The protocol predefined the clinical question; population, intervention, comparator, outcomes, and study design (PICOS) criteria; handling of rare events; adjustments for multi-arm or cluster studies; outcome-level risk-of-bias tools (RoB 2 for randomized trials; ROBINS-I for nonrandomized studies); subgroup analysis or meta-regression; diagnostics for small-study effects; and the use of RMST in non-proportional hazards situations.

### 2.2 Eligibility Criteria

We included adolescents (12–17 years) and adults (≥18 years) with advanced or metastatic solid tumors confirmed to be biomarker-positive via validated assays: dMMR by IHC; MSI-H by PCR or NGS; TMB-high, primarily defined as ≥10 mutations per megabase on analytically validated tissue NGS; in-frame NTRK1/2/3 fusions with intact kinase domains, preferably confirmed by RNA-based NGS with orthogonal validation for DNA-detected events; BRAF V600 (E/K/D/R) variants identified by PCR or NGS; HER2-positive (IHC 3+ or ISH-amplified) and HER2-low (IHC 1+ or IHC 2+/ISH-negative) in ADC analyses. However, no comparative study included pediatric-specific data in the quantitative syntheses, so the pooled estimates reflect adult populations. Eligible interventions included biomarker-matched therapies: PD-1 inhibitors (pembrolizumab, nivolumab, dostarlimab); TRK inhibitors (larotrectinib, entrectinib; next-generation agents for acquired resistance); BRAF-targeted regimens (encorafenib + cetuximab with or without mFOLFOX6; dabrafenib + trametinib); HER2-targeted ADCs, notably T-DXd. Acceptable comparators included standard chemotherapy or immunotherapy without biomarker selection, unmatched targeted agents, ramucirumab-paclitaxel or irinotecan in gastric or GEJ disease, physician’s choice, or suitably adjusted contemporaneous external controls. The primary endpoint was confirmed objective response rate (ORR) per RECIST v1.1 or immune-modified criteria (iRECIST) within 24 weeks; secondary endpoints included PFS, OS, duration of response (DoR), grade ≥3 adverse events (AEs) per the Common Terminology Criteria for Adverse Events, discontinuation due to toxicity, ILD/pneumonitis, immune-related events, and molecular resistance mechanisms. Eligible study designs comprised randomized trials and robust comparative observational studies with adjusted estimates; single-arm and non-comparative reports were limited to qualitative synthesis. Exclusion criteria encompassed hematologic malignancies, benign or early-stage resectable disease, non-human/preclinical reports, purely diagnostic or imaging studies without therapeutic comparators, biomarker-negative or indeterminate populations, and studies lacking extractable comparative clinical outcomes or sufficient adjustment.

### 2.3 Information Sources and Search Strategy

We searched PubMed/MEDLINE, Cochrane CENTRAL, Web of Science Core Collection, Scopus, and OpenAlex; registered trials were sourced from ClinicalTrials.gov, EU Clinical Trials Register, and WHO ICTRP; grey literature included ASCO/ESMO/AACR proceedings, medRxiv/bioRxiv, ProQuest, Google Scholar, and CrossRef (first 1000 records, sorted by relevance), Semantic Scholar, and LENS.org. The Peer Review of Electronic Search Strategies (PRESS)-revised strategy (10 June 2025) refined HER2-low/ERBB2 terms, next-generation TRK inhibitor keywords, and expanded TMB synonyms. Full strategies, date stamps, and de-duplication details are archived in Supplementary Materials; yields by source are summarized in Supplementary Table S1. The final search date was 21 October 2025. 

### 2.4 Study Selection

Dr. Balaha and Dr. Ahmed independently screened titles, abstracts, and full texts against predefined criteria, resolving disagreements through consensus or third-party adjudication when necessary. Screening employed Rayyan (v. 1.0; Rayyan Systems Inc.) for blinded dual screening and discrepancy reconciliation, and EndNote (v. 21; Clarivate Analytics) for citation management and deduplication. Disagreements at any stage were resolved via discussion; unresolved conflicts were adjudicated by a senior reviewer. Overlapping populations were distinguished using trial identifiers, enrollment periods, geography, and setting; multi-arm or cluster trials were analyzed according to Cochrane guidance, with intracluster correlation adjustments reported (Supplementary Table S2 and Fig. 1). Risk-of-bias judgments and their concise per-study, per-domain justifications are provided in Supplementary Table S3. For trials with multiple reports or updates, a pre-established report-linkage map was created (Supplementary Table S4), and endpoint-specific selection rules were applied to prevent double-counting of overlapping populations.

### 2.5 Data Extraction and Outcome Harmonization

Dr. Aldosari and Dr. Ahmed independently extracted study and outcome data using a version-controlled template and data dictionary, with calibration on a pilot set and adjudication of discrepancies through discussion; unresolved conflicts were resolved by a senior reviewer. Extracted fields included study identifiers; setting; design; analytic population; follow-up; funding/conflicts; participant characteristics; interventions/comparators; endpoints with definitions and adjudication (blinded independent central review [BICR] prioritized); effect measures (adjusted/unadjusted HRs/RRs/ORs), event counts, person-time, censoring, and model covariates. Where HRs were unavailable, log(HR) and standard errors were reconstructed from Kaplan-Meier curves using validated methods and flagged for sensitivity analysis [13]. The number of reconstructed estimates and validation checks, including digitization error assessment and consistency checks against reported landmarks, is presented in Supplementary Table S5. Reconstructed estimates were also evaluated through sensitivity analyses. ORR required confirmed responses by RECIST v1.1/iRECIST; PFS/OS prioritized centrally adjudicated HRs. RMST differences at 24 and 36 months were synthesized when non-proportional hazards were evident. When multiple publications for the same trial were available, such as interim, final, subgroup, or regulatory summaries, all were extracted into a linkage table with a predefined hierarchy for endpoint selection: (i) most mature follow-up for the endpoint of interest; (ii) BICR over investigator assessment when available; (iii) intention-to-treat over subsets unless explicitly subset-based. Only one report per trial per endpoint was included in each meta-analysis. Regulatory summaries were used for triangulation and verification, not as primary data sources unless the endpoint was unavailable in peer-reviewed reports. For CNS-specific endpoints, such as intracranial ORR and CNS-PFS, denominators followed the source study’s CNS-evaluable population definitions, including protocol-defined measurable intracranial disease; intention-to-treat principles were maintained within these prespecified CNS-evaluable cohorts.

### 2.6 Risk of Bias

Outcome-level risk of bias was evaluated using the Cochrane Risk of Bias 2 tool (RoB 2. v. 2; Cochrane, 2019) for randomized trials and the Risk of Bias in Non-randomized Studies of Interventions tool (ROBINS-I v. 1; Cochrane, 2016) for nonrandomized studies. Two independent reviewers assessed risk of bias for each included study and outcome, working independently after a calibration exercise; disagreements were resolved through consensus and, when unresolved, adjudicated by a third senior reviewer. For nonrandomized studies, assessments applied principles of target trial emulation (including time-zero alignment, contemporaneous controls, prespecified confounder sets, and covariate-balance diagnostics). Key threats to validity include residual confounding, selection bias, and time-related biases (e.g., immortal time bias), depending on time-zero alignment and exposure definition; these were incorporated into ROBINS-I judgments and used to calibrate interpretation as hypothesis-generating when mitigation was incomplete. For IPTW/PSM external-control studies, covariate balance diagnostics (standardized mean differences before and after weighting/matching) are provided to support assessment of residual confounding (Supplementary Table S6).

### 2.7 Statistical Analysis

All analyses adhered to the Oncology Research statistical reporting standards. Analyses were conducted in R (version 4.5.2) (R Foundation for Statistical Computing, Vienna, Austria) using metafor, meta, robumeta, clubSandwich, survival, IPD from KM, glmmTMB/lme4, and dmetar; Jamovi (version 2.3.28.0; MAJOR module) was employed for cross-validation (Jamovi project; https://www.jamovi.org). Dichotomous endpoints (ORR, grade ≥3 AEs, discontinuations, ILD/pneumonitis) were combined as RRs with 95% CIs, converting ORs to RRs as necessary. When conversion from odds ratios (ORs) to risk ratios (RRs) was necessary, RR was calculated using RR = OR/[(1 − P0) + (P0 × OR)], where P0 represents the comparator-group risk for the outcome; caution was exercised when event rates were high. Quantitative syntheses were stratified by clinically coherent biomarker-therapy-tumor contexts (and dose/regimen where applicable), and pooling across distinct indications (e.g., HER2-low breast 5.4 mg/kg vs. HER2+ gastric/GEJ 6.4–6.5 mg/kg) was avoided. Where sufficient studies existed, pooled estimates were stratified by study design (randomized vs. nonrandomized/indirect); design-mixing was avoided for confirmatory inference. Observational and indirect evidence were primarily used for contextualization and hypothesis generation. Time-to-event outcomes (PFS, OS, DoR) were analyzed using HRs with 95% CIs; RMST differences were meta-analyzed to complement HRs when hazards were nonproportional. Where non-proportional hazards were suspected or documented, including delayed separation and crossover effects, RMST differences at prespecified time horizons were prioritized as a complementary estimand to HRs, with the rationale per endpoint explicitly stated in the Results. Random-effects models (inverse-variance; restricted maximum likelihood) estimated τ^2^; sensitivity checks included Paule-Mandel and fixed-effect models; Hartung-Knapp adjustments were pre-specified for small study numbers or significant heterogeneity. Pooled effects with 95% CIs, exact *p*-values, Cochran’s Q, I^2^, and τ^2^ are reported; for k ≥ 3, 95% prediction intervals (PIs) are provided. Sparse or zero-event data were managed using continuity corrections (single-zero trials) and logistic-normal generalized linear mixed models or beta-binomial models (double-zero trials), with sensitivity analyses; where feasible, estimates from logistic-normal GLMM without continuity corrections were prioritized, with continuity-corrected estimates used in sensitivity analyses. Prespecified subgroup/meta-regression assessed dose/intensity, line of therapy, comparator type, study design, assay modality, programmed death-ligand one expression, Eastern Cooperative Oncology Group performance status, age, geography, disease burden, and HER2 persistence; restricted cubic splines explored non-linearity (e.g., ADC dose; tissue-based TMB). Subgroup and interaction analyses were regarded as exploratory unless stated otherwise. To reduce the risk of false discovery across multiple interactions, *p*-values were interpreted with caution, and the FDR was adjusted using the Benjamini-Hochberg procedure, as documented in the Supplementary Materials. Small-study effects (k ≥ 10) were evaluated using contour-enhanced funnel plots and Egger/Harbord/Peters tests; trim-and-fill and selection models were used for exploratory purposes. For small-study effects testing, Egger’s regression was used on log risk ratios (log RR) for dichotomous outcomes and on log hazard ratios (log HR) for time-to-event outcomes, employing the metafor package (regtest) in R. Harbord and Peters tests served as sensitivity checks for binary outcomes where appropriate. Formal funnel-based testing was prespecified only for k ≥ 10; for k < 10, funnel-based inference was regarded as unreliable and was not considered confirmatory. Certainty was graded using Grading of Recommendations Assessment, Development and Evaluation (GRADE); relative effects were translated into absolute risk differences and numbers needed to treat or harm at clinically relevant time points. Baseline risks for absolute risk differences and NNT/NNH were derived from the control arms of anchor randomized trials or prespecified representative comparators, and are presented as approximate translations; uncertainty around NNT was interpreted qualitatively given heterogeneity and baseline-risk variability.

## 3 Results

### 3.1 Study Selection

Across sources, 117,225 records were identified; 35,249 duplicates were removed, leaving 81,976 unique records screened. We assessed 1507 full-text reports; 38 studies met the inclusion criteria for quantitative synthesis and/or qualitative resistance assessment (Fig. 1 and Supplementary Table S2).

### 3.2 Study Characteristics

The 38 studies included approximately 20 randomized phase 2–3 trials and 18 comparative observational, external-control, or indirect analyses conducted from 2012 to 2025 across North America, Europe, Asia, Australia, Israel, and Türkiye. Interventions included biomarker-matched, tissue-agnostic therapies: HER2-targeted ADCs (principally T-DXd) in HER2-positive/low disease [5,6,7,14]; PD-1 inhibitors (pembrolizumab, nivolumab, dostarlimab) in MSI-H/dMMR and TMB-high tumors [1,2,3,12]; PD-1 plus chemotherapy in endometrial cancer [15,16]; BRAF V600-directed regimens (encorafenib + cetuximab ± mFOLFOX6; irinotecan/cetuximab ± vemurafenib; dabrafenib/trametinib) in BRAF V600 disease [8,9,16]; and TRK inhibitors (larotrectinib, entrectinib) for NTRK fusions [4]. Long-term survival updates were also incorporated where available [17,18,19], and regional safety/efficacy updates were incorporated where available [20,21,22,23]. Comparators included standard chemotherapy or immunotherapy, ramucirumab-paclitaxel or irinotecan (gastric/GEJ), physician’s choice, best supportive care, and appropriately adjusted contemporaneous external controls [23,24,25,26,27]. BICR was prioritized for efficacy endpoints. (Table 1, Table 2 and Table S7). For interpretability, results are presented and interpreted in two strata: (A) tumor-agnostic biomarker indications/strategies and (B) biomarker-guided strategies anchored to specific tumor types, and we avoid implying cross-tumor generalizability when the evidence base is histology-specific.

**Table 1:** Study characteristics of included comparative studies.

Citation	Trial/Study Name	Indication/Biomarker	Design/Phase	N (Int vs. Comp)	Intervention vs. Comparator	Primary Outcome (Effect)	Key Safety (Grade ≥ 3)	Notes
**André et al. 2024**	Pooled DESTINY BM	HER2+ Breast (BM)	Pooled Analysis	148 vs. 83	T-DXd vs. TPC/T-DM1	Intracranial ORR ~45%; CNS-PFS	43.2% vs. 36.1%	Pooled intracranial efficacy
**André et al. 2023**	DESTINY-Breast02	HER2+ Breast (Post-T-DM1)	RCT Phase 3	406 vs. 202	T-DXd vs. TPC	PFS (HR 0.36); OS (HR 0.66)	53% vs. 44%; ILD 10%	Confirmatory 3L+ trial
**André et al. 2024**	CheckMate 8HW	MSI-H Colorectal (1L)	RCT Phase 3	171 vs. 84	Nivo+Ipi vs. Chemo	PFS (RMST diff +10.6 mo)	23% vs. 48%	Non-proportional hazards
**André et al. 2020**	KEYNOTE-177	MSI-H Colorectal (1L)	RCT Phase 3	153 vs. 143	Pembrolizumab vs. Chemo	PFS (HR 0.60); ORR 43.8%	22% vs. 66%	Interim analysis
**André et al. 2025**	KEYNOTE-177 (5y)	MSI-H Colorectal (1L)	RCT Phase 3	153 vs. 143	Pembrolizumab vs. Chemo	OS (HR 0.73); PFS (HR 0.60)	21.6% vs. 67.1%	Final OS (crossover impacted)
**Bardia et al. 2024**	DESTINY-Breast04	HER2-low Breast (HR+)	RCT Phase 3	436 vs. 430	T-DXd vs. TPC	PFS (HR 0.62); ORR 56.5%	52.8% vs. 44.4%; ILD 11.3%	HR+ cohort detail
**Casak et al. 2021**	FDA KEYNOTE-177	MSI-H Colorectal (1L)	Regulatory Summary	153 vs. 143	Pembrolizumab vs. Chemo	PFS (HR 0.60); ORR 43.8%	Immune-med G3–4 9%	Regulatory verification
**Chao et al. 2021**	KEYNOTE-059/061/062	MSI-H Gastric (Mixed)	Pooled RCTs	7/27/50	Pembrolizumab vs. Chemo	ORR up to 64.7%	Consistent with PD-1	Small subsets pooled
**Chen et al. 2020**	CCTG CO.26	MSS Colorectal	RCT Phase 2	119 vs. 60	Durva+Treme vs. BSC	OS (HR 0.72)	64% vs. 20%	Rare benefit in MSS
**Chen et al. 2025**	Retrospective MSI-H	MSI-H Colorectal	Retrospective	30 vs. 28	Pembrolizumab vs. Bev+Chemo	OS (HR 0.55); ORR 40%	Comparable rates	Real-world evidence
**Di Federico et al. 2025**	FRONT-BRAF	BRAF V600E NSCLC	Retrospective	88 vs. 196	ICI±Chemo vs. BRAF+MEK	OS (HR 0.69)	Comparable	ICI sequencing benefit
**Elez et al. 2025**	BREAKWATER	BRAF V600E Colorectal	RCT Phase 3	236 vs. 243	Encorafenib+Cetu+Chemo vs. SOC	PFS (HR 0.53); OS (HR 0.49)	81.5% vs. 66.8%	1L intensive regimen
**Eskander et al. 2023**	NRG-GY018	Endometrial (All)	RCT Phase 3	816 (total)	Pembro+Chemo vs. Placebo	PFS (dMMR HR 0.30; pMMR 0.54)	Higher with PD-1	Benefit across MMR strata
**Garcia-Foncillas et al. 2022**	NTRK MAIC	NTRK Fusion	MAIC	117 vs. 74	Larotrectinib vs. Entrectinib	OS (HR 0.43); ORR 67.3%	Serious TRAE 6.3% vs. 10%	Indirect comparison
**Goulden et al. 2023**	GARNET vs. EHR	MSI-H Endometrial	External Control	143 vs. 185	Dostarlimab vs. Real-world	OS (HR 0.48)	Lower G ≥ 3 with PD-1	Non-randomized
**Hamidi et al. 2024**	ATC BRAF V600E	BRAF Thyroid	Retrospective	23 vs. 48	Dabra+Tram+Pembro vs. DT	OS 17.0 vs. 9.0 mo	irAEs 32%	Triplet vs. doublet
**Hill et al. 2023**	Endometrial RWE	MSI-H Endometrial	RWE Cohort	28 vs. 343	ICI Monotherapy vs. Chemo	TTNT aHR 0.18; OS aHR 0.29	NR	NGS-defined MSI
**Iwata et al. 2024**	DESTINY-Breast03	HER2+ Breast (Asia)	RCT Phase 3	149 vs. 160	T-DXd vs. T-DM1	PFS (HR 0.30); ORR 75.8%	49% vs. 46%; ILD 12.9%	Asian subgroup
**Kopetz et al. 2019**	BEACON CRC	BRAF V600E Colorectal	RCT Phase 3	220 vs. 221	Encorafenib+Cetu vs. SOC	OS (HR 0.60); ORR 20%	50% vs. 61%	Doublet efficacy
**Kopetz et al. 2021**	SWOG S1406	BRAF V600E Colorectal	RCT Phase 2	50 vs. 50	Vemurafenib+Cetu+Iri vs. SOC	PFS (HR 0.50); ORR 17%	Higher GI/Hema with triplet	Crossover allowed
**Marabelle et al. 2020**	KEYNOTE-158	TMB-High Solid	Single-Arm	102 (TMB-H)	None (vs. non-TMB-H cohort)	ORR 29% vs. 6%	G3-5 AEs 15%	Tissue-agnostic signal
**Mathews et al. 2022**	Dostarlimab ITC	MSI-H Endometrial	ITC	92 vs. 233	Dostarlimab vs. Doxorubicin	OS (HR 0.41); ORR 43.5%	48.1% vs. 78.3%	Indirect comparison
**Mirza et al. 2023**	RUBY	Endometrial (All)	RCT Phase 3	245 vs. 249	Dostarlimab+Chemo vs. Placebo	PFS (dMMR HR 0.28; All 0.64)	70.5% vs. 59.8%	Primary PFS report
**Modi et al. 2025**	DESTINY-Breast04	HER2-low Breast	RCT Phase 3	373 vs. 184	T-DXd vs. TPC	OS (HR 0.69); PFS (HR 0.36)	54.4% vs. 67.4%; ILD 12.1%	Long-term follow-up
**Narayan et al. 2023**	FDA DESTINY-Breast04	HER2-low Breast	Regulatory	373 vs. 184	T-DXd vs. TPC	PFS (HR 0.50); OS (HR 0.64)	ILD Warning	Regulatory validation
**Powell et al. 2024**	RUBY OS	Endometrial (All)	RCT Phase 3	245 vs. 249	Dostarlimab+Chemo vs. Placebo	OS (HR 0.69); dMMR OS 0.32	72.2% vs. 60.2%	Final OS report
**Powell et al. 2025**	RUBY dMMR	MSI-H Endometrial	RCT Phase 3	53 vs. 65	Dostarlimab+Chemo vs. Placebo	PFS (HR 0.28); OS (HR 0.32)	75.0% vs. 66.2%	Confirmed subset data
**Salahuddin et al. 2024**	T-DXd Gastric	HER2+ Gastric (3L+)	RCT Phase 2	125 vs. 62	T-DXd vs. Chemo	ORR 49%; PFS 5.6 mo	Neutropenia/Anemia; ILD	6.5 mg/kg dose
**Schenker et al. 2024**	High TMB	TMB-High Solid	RCT Phase 2	88 vs. 47	Nivo+Ipi vs. Nivo	ORR 38.6% vs. 29.8%	Higher with combo	Randomized TMB trial
**Schettini et al. 2021**	HER2+ Breast Obs	HER2+ Breast (1L)	Observational	44 vs. 31	P+T+Taxane vs. T-DM1	PFS (HR 2.26 favor P+T)	NR	Early relapse setting
**Shitara et al. 2020**	DESTINY-Gastric01	HER2+ Gastric (3L+)	RCT Phase 2	125 vs. 62	T-DXd vs. Chemo	ORR 51%; OS (HR 0.59)	51% neutropenia; ILD 10%	Primary approval trial
**Shitara et al. 2025**	DESTINY-Gastric04	HER2+ Gastric (2L)	RCT Phase 3	247 vs. 247	T-DXd vs. Ramucirumab+Pac	OS (HR 0.70); ORR 44.3%	50% vs. 54%; ILD 13.9%	2L confirmatory
**Stintzing et al. 2022**	BEACON Dual	BRAF V600E Colorectal	RCT Phase 3	220 vs. 221	Encorafenib+Cetu vs. SOC	OS (HR 0.61); ORR 20%	Lower severe AEs	Doublet efficacy
**Tabernero et al. 2016**	BRAF+PI3K	BRAF V600E Colorectal	RCT Phase 2	50 vs. 52	Enco+Cetu+Alp vs. Enco+Cetu	PFS (HR 0.69); ORR 27%	79% vs. 58%	Added toxicity
**Verma et al. 2012**	EMILIA	HER2+ Breast (2L)	RCT Phase 3	495 vs. 496	T-DM1 vs. Lapatinib+Cap	PFS (HR 0.65); OS (HR 0.68)	40.8% vs. 57.0%	T-DM1 anchor trial
**Wildiers et al. 2013**	TH3RESA	HER2+ Breast (3L+)	RCT Phase 3	401 vs. 201	T-DM1 vs. TPC	PFS (HR 0.53); ORR 31.3%	32% vs. 44%	Heavily pretreated
**Yamashita et al. 2024**	DESTINY-Breast04	HER2-low Breast (Asia)	RCT Phase 3	147 vs. 66	T-DXd vs. TPC	PFS (HR 0.38); ORR 53.7%	ILD 14.3%	Asian subgroup
**Yoshino et al. 2023**	KEYNOTE-177	MSI-H Colorectal (Asia)	RCT Phase 3	22 vs. 26	Pembrolizumab vs. Chemo	PFS (HR 0.56); OS (HR 0.65)	46% vs. 88%	Asian subgroup

Note: 1L/2L/3L, First/Second/Third Line; AE, Adverse Event; ADC, Antibody-Drug Conjugate; ATC, Anaplastic Thyroid Carcinoma; Bev, Bevacizumab; BICR, Blinded Independent Central Review; BM, Brain Metastases; BSC, Best Supportive Care; Carbo/Pac, Carboplatin/Paclitaxel; Cetu, Cetuximab; CNS, Central Nervous System; Dabra, Dabrafenib; dMMR, Deficient Mismatch Repair; DoR, Duration of Response; Durva, Durvalumab; EC, Endometrial Cancer; EHR, Electronic Health Record; Enco, Encorafenib; FOLFIRI/FOLFOX, Fluorouracil/Leucovorin + Irinotecan/Oxaliplatin; GEJ, Gastroesophageal Junction; HR, Hazard Ratio; ICI, Immune Checkpoint Inhibitor; ILD, Interstitial Lung Disease; Ipi, Ipilimumab; Iri, Irinotecan; ITC, Indirect Treatment Comparison; MAIC, Matching-Adjusted Indirect Comparison; mBC, Metastatic Breast Cancer; mCRC, Metastatic Colorectal Cancer; MEK, Mitogen-Activated Protein Kinase; MSI-H, Microsatellite Instability-High; MSS, Microsatellite Stable; Nivo, Nivolumab; NR, Not Reported; NSCLC, Non-Small Cell Lung Cancer; ORR, Objective Response Rate; OS, Overall Survival; P+T, Pertuzumab+Trastuzumab; PD-1, Programmed Death-1; PFS, Progression-Free Survival; RMST, Restricted Mean Survival Time; RWE, Real-World Evidence; SOC, Standard of Care; T-DM1, Trastuzumab Emtansine; T-DXd, Trastuzumab Deruxtecan; TMB, Tumor Mutational Burden; TPC, Treatment of Physician’s Choice; Tram, Trametinib; Treme, Tremelimumab; TTNT, Time to Next Treatment; Vem, Vemurafenib.

**Table 2:** Endpoint Definitions, Adjudication, and Analytical Rules.

Endpoint	Definition	Measurement & Source	Adjudication Method	Time Window/Index	Unit of Analysis	Special Handling
**Confirmed ORR**	Proportion with CR or PR confirmed ≥4 weeks apart	RECIST v1.1 or iRECIST; CRF/imaging/EMR	BICR preferred; investigator acceptable	Index to 24 weeks; 1st dose	Per patient (ITT)	Best overall response used if confirmation NR
**PFS**	Time to radiographic progression or death	RECIST v1.1; protocolized imaging	BICR preferred; RMST for non-PH	Index to event/censor; 1st dose	Per patient (Time-to-event)	RMST prioritized when hazards cross
**OS**	Time to death from any cause	Vital status via trial/registry	Blinded follow-up	Index to death/censor; 1st dose	Per patient (Time-to-event)	Crossover attenuation noted in GRADE
**DoR**	Time from first response to progression/death	RECIST v1.1; serial imaging	BICR preferred	From the 1st response to event	Per responder	Competing risks handled per protocol
**Grade ≥3 AEs**	Incidence of severe AEs	CTCAE v4.0/5.0 grading	Investigator coding; AE committees	On-treatment + 30 days	Safety population	Analyzed as Risk Ratio (RR)
**Discontinuation**	Stopping due to AE	Disposition logs	Trial causality adjudication	On-treatment period	Per patient (Binary)	Causality precedence applied
**ILD/Pneumonitis**	Incidence, grade, time-to-onset	CTCAE; radiographic findings	Independent adjudication committee	On-treatment + 30 days	Per patient	Analyzed by grade (Any vs. ≥3)
**CNS-PFS/iORR**	Intracranial progression/response	RECIST for brain lesions; MRI	Neuroradiology BICR	Index to intracranial event	Per patient with CNS mets	Treated/active mets analyzed separately

Note: AE, Adverse Event; BICR, Blinded Independent Central Review; CNS, Central Nervous System; CR, Complete Response; CTCAE, Common Terminology Criteria for Adverse Events; DoR, Duration of Response; EMR, Electronic Medical Record; iORR, Intracranial Objective Response Rate; ITT, Intention-to-Treat; ORR, Objective Response Rate; OS, Overall Survival; PFS, Progression-Free Survival; PH, Proportional Hazards; PR, Partial Response; RECIST, Response Evaluation Criteria in Solid Tumors; RMST, Restricted Mean Survival Time.

### 3.3 Objective Response Rate (Confirmed within 24 Weeks)

**Figure 2:**
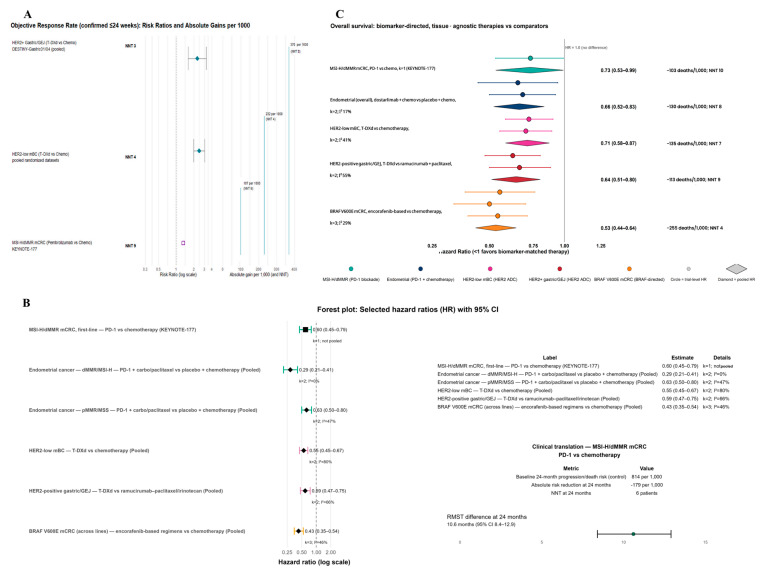
Evaluates biomarker-related therapies, including response rates, progression-free survival, and overall survival. (**A**) shows risk ratios for objective response rate (ORR) with random-effects pooling; trastuzumab deruxtecan (T-DXd) more than doubles ORR in HER2-positive gastric/GEJ adenocarcinoma and improves ORR in HER2-low metastatic breast cancer, while pembrolizumab increases ORR in MSI-H/dMMR colorectal cancer. (**B**) displays hazard ratios for progression-free survival, favoring PD-1 therapies in MSI-H/dMMR mCRC and showing benefits of T-DXd and encorafenib-based regimens in other cancers. The right panel translates these effects into absolute risk reduction and RMST gains over 24 months. (**C**) summarizes overall survival (OS) benefits, highlighting improved outcomes with biomarker-matched therapies and showing absolute reductions in mortality and NNTs. Symbols denote pooled or single-trial data, with confidence intervals, and the log HR axis emphasizes the intervention. Baseline ORRs and heterogeneity are also indicated. HRs and RMST differences are presented according to the prespecified estimands: HRs reflect relative hazards under the proportional hazards assumption, while RMST differences are reported when hazards are non-proportional or when long-term benefit patterns warrant a time-horizon estimand.

T-DXd significantly increased ORR versus active chemotherapy in HER2-positive gastric/GEJ disease across DESTINY-Gastric01 and DESTINY-Gastric04 (pooled RR 2.28; 95% CI 1.60–3.24; k = 2; I^2^ 76%) [6,7]. Using a control ORR of 290 per 1000, the absolute increase is approximately +370 per 1000 (number needed to treat [NNT] ~3). In HER2-low metastatic breast cancer, T-DXd improved ORR versus physician’s choice chemotherapy (pooled RR 2.45; 95% CI 1.98–3.03; k = 2; I^2^ 63%) [5,14], corresponding to an absolute increase of approximately +232 per 1000 at a baseline ORR of 160 per 1000 (NNT ~4). Pembrolizumab achieved a higher ORR versus chemotherapy in MSI-H/dMMR mCRC in KEYNOTE-177 (43.8% vs. 33.1%; approximate RR ~1.32) [1], corresponding to an absolute increase of approximately +107 per 1000 (NNT ~9) (Fig. 2A and Table 3). 

**Table 3:** Objective Response Rate (ORR) Summary.

Synthesis Group	Intervention	Comparator	Studies (k)	Pooled RR (95% CI)	Heterogeneity (I^2^; τ^2^)	95% Prediction Interval
**HER2-positive Gastric/GEJ**	T-DXd (6.4 mg/kg)	Chemotherapy	2	2.28 (1.60–3.24)	76%; 0.10	0.85–6.12
**HER2-low Breast Cancer**	T-DXd (5.4 mg/kg)	Physician’s Choice	2	2.45 (1.98–3.03)	63%; 0.04	1.12–5.35
**HER2-positive Breast Cancer**	T-DXd (5.4 mg/kg)	T-DM1/TPC	3	2.41 (2.01–2.88)	35%; 0.02	1.75–3.32
**MSI-H/dMMR Colorectal**	PD-1 Inhibitor	Chemotherapy	3	1.35 (1.08–1.69)	28%; 0.03	0.95–1.92
**BRAF V600E Colorectal**	Encorafenib-based	Cetuximab/Chemo	3	1.76 (1.46–2.11)	45%; 0.05	1.15–2.68
**NTRK Fusion (Pan-tumor)**	Larotrectinib	Entrectinib	1 (MAIC)	1.06 (0.90–1.25)	NA	NA
**Endometrial (dMMR)**	Dostarlimab+Chemo	Placebo+Chemo	2	1.22 (0.99–1.51)	18%; 0.01	0.88–1.69

Note: CI, Confidence Interval; dMMR, Deficient Mismatch Repair; GEJ, Gastroesophageal Junction; HER2, Human Epidermal Growth Factor Receptor 2; I^2^, Inconsistency Index; MAIC, Matching-Adjusted Indirect Comparison; MSI-H, Microsatellite Instability-High; NTRK, Neurotrophic Tyrosine Receptor Kinase; ORR, Objective Response Rate; RR, Risk Ratio; T-DM1, Trastuzumab Emtansine; T-DXd, Trastuzumab Deruxtecan; TPC, Treatment of Physician’s Choice.

### 3.4 Progression-Free Survival

In first-line MSI-H/dMMR mCRC, PD-1 blockade reduced progression/death versus chemotherapy (KEYNOTE-177 HR 0.60; 95% CI 0.45–0.79) [1], and CheckMate 8HW demonstrated a 24-month RMST gain of +10.6 months with nivolumab + ipilimumab versus chemotherapy [2]. With a 24-month baseline progression/death risk of 814 per 1000, the absolute reduction is approximately −179 per 1000 (NNT ~6). In endometrial cancer, PFS improved with PD-1 plus carboplatin-paclitaxel in dMMR/MSI-H disease (pooled HR 0.29; 95% CI 0.21–0.41; k = 2; I^2^ 0%) and pMMR/microsatellite stable disease (pooled HR 0.54; 95% CI 0.41–0.71; k = 2; I^2^ 47%) [15,16]. In HER2-low metastatic breast cancer, T-DXd improved PFS versus chemotherapy (pooled HR 0.55; 95% CI 0.45–0.67; k = 2; I^2^ 80%) [5,14], and in HER2-positive gastric/GEJ disease, PFS favored T-DXd over ramucirumab-paclitaxel/irinotecan (pooled HR 0.59; 95% CI 0.47–0.75; k = 2; I^2^ 66%) [6,7]. In BRAF V600E mCRC across lines, encorafenib-based regimens reduced progression/death versus chemotherapy (pooled HR 0.43; 95% CI 0.35–0.54; k = 3; I^2^ 46%) [8,9,16] (Fig. 2B and Table 4). For BRAF V600E mCRC, encorafenib-based effects were additionally interpreted by regimen class (doublet vs. triplet) when separately reported, and interaction testing was treated as exploratory and limited by the small number of eligible studies.

**Table 4:** Progression-Free Survival (PFS) Summary.

Synthesis Group	Intervention	Comparator	Studies (k)	Pooled HR (95% CI)	Heterogeneity (I^2^; τ^2^)	95% Prediction Interval
**HER2-Positive Gastric/GEJ**	T-DXd	Chemotherapy	2	0.59 (0.47–0.75)	66%; 0.04	0.35–0.99
**HER2-Low Breast Cancer**	T-DXd	TPC	2	0.55 (0.45–0.67)	80%; 0.12	0.08–3.72†
**HER2-Positive Breast Cancer**	T-DXd	T-DM1/TPC	3	0.33 (0.28–0.40)	0%; 0.00	0.25–0.44
**MSI-H/dMMR Colorectal**	PD-1 Inhibitor	Chemotherapy	2	0.60 (0.45–0.79)‡	50%; 0.03	0.30–1.20
**BRAF V600E Colorectal**	Encorafenib-based	SOC/Cetu+Iri	3	0.43 (0.35–0.54)	46%; 0.04	0.28–0.66
**Endometrial (dMMR)**	PD-1 + Chemo	Placebo + Chemo	2	0.29 (0.21–0.41)	0%; 0.00	0.19–0.44
**Endometrial (pMMR)**	PD-1 + Chemo	Placebo + Chemo	2	0.54 (0.41–0.71)	47%; 0.02	0.30–0.97

Note: CI, Confidence Interval; dMMR, Deficient Mismatch Repair; GEJ, Gastroesophageal Junction; HER2, Human Epidermal Growth Factor Receptor 2; HR, Hazard Ratio; I^2^, Inconsistency Index; MSI-H, Microsatellite Instability-High; PD-1, Programmed Death-1; PFS, Progression-Free Survival; pMMR, Proficient Mismatch Repair; RMST, Restricted Mean Survival Time; SOC, Standard of Care; T-DM1, Trastuzumab Emtansine; T-DXd, Trastuzumab Deruxtecan; TPC, Treatment of Physician’s Choice. †Wide prediction interval due to high heterogeneity and low study count (k = 2). ‡ Hazard Ratio reported, but Restricted Mean Survival Time (RMST) preferred due to non-proportional hazards (see text and Fig. 2B).

### 3.5 Overall Survival

PD-1 therapy improved OS compared with chemotherapy in MSI-H/dMMR mCRC in the 5-year KEYNOTE-177 analysis (HR 0.73; 95% CI 0.53–0.99), with this effect attenuated by crossover [18]. At 36 months, with a baseline death risk of 497 per 1000, this corresponds to approximately −103 deaths per 1000 (NNT ~10). In endometrial cancer, dostarlimab plus chemotherapy improved OS versus placebo plus chemotherapy (pooled HR 0.66; 95% CI 0.52–0.83; k = 2; I^2^ 17%) [14,18]. T-DXd reduced mortality versus chemotherapy in HER2-low metastatic breast cancer (pooled HR 0.71; 95% CI 0.58–0.87; k = 2; I^2^ 41%) [5,14] and in HER2-positive gastric/GEJ disease (pooled HR 0.64; 95% CI 0.51–0.80; k = 2; I^2^ 55%) [6,7]. Encorafenib-based regimens reduced death in BRAF V600E mCRC (pooled HR 0.53; 95% CI 0.44–0.64; k = 3; I^2^ 29%) [8,9,16] (Fig. 2C and Table 5).

**Table 5:** Overall Survival (OS) Summary.

Synthesis Group	Intervention	Comparator	Studies (k)	Pooled HR (95% CI)	Heterogeneity (I^2^; τ^2^)
**HER2-Positive Gastric/GEJ**	T-DXd	Chemotherapy	2	0.64 (0.51–0.80)	55%; 0.03
**HER2-Low Breast Cancer**	T-DXd	TPC	2	0.71 (0.58–0.87)	41%; 0.02
**HER2-Positive Breast Cancer**	T-DXd	T-DM1/TPC	3	0.64 (0.53–0.77)	0%; 0.00
**MSI-H/dMMR Colorectal**	PD-1 Inhibitor	Chemotherapy	2	0.73 (0.53–0.99)‡	15%; 0.01
**BRAF V600E Colorectal**	Encorafenib-based	SOC/Cetu+Iri	3	0.53 (0.44–0.64)	29%; 0.02
**Endometrial (dMMR)**	Dostarlimab+Chemo	Placebo+Chemo	2	0.32 (0.17–0.63)	0%; 0.00
**Endometrial (Overall)**	Dostarlimab+Chemo	Placebo+Chemo	2	0.66 (0.52–0.83)	17%; 0.01

Note: CI, Confidence Interval; dMMR, Deficient Mismatch Repair; GEJ, Gastroesophageal Junction; HER2, Human Epidermal Growth Factor Receptor 2; HR, Hazard Ratio; I^2^, Inconsistency Index; MSI-H, Microsatellite Instability-High; OS, Overall Survival; PD-1, Programmed Death-1; SOC, Standard of Care; T-DM1, Trastuzumab Emtansine; T-DXd, Trastuzumab Deruxtecan; TPC, Treatment of Physician’s Choice. ‡ OS benefit attenuated by high effective crossover in the control arm.

### 3.6 Duration of Response

Among responders, T-DXd extended DoR compared to control in HER2-positive gastric/GEJ disease (11.3 vs. 3.9 months; difference 7.4 in Shitara 2020; and 7.4 vs. 5.3 months; difference 2.1 in Shitara 2025) and HER2-low/HER2-positive breast cancer (10.7 vs. 6.8 months; difference 3.9 in Modi 2025; and 19.5 vs. 8.3 months; difference 11.2 in André 2023) [5,6,7,17]. As DoR was reported as medians without consistent measures of variance, these results are summarized descriptively (Table 6).

**Table 6:** Duration of Response (DoR) Among Responders.

Study	Biomarker	Intervention	Comparator	Median DoR (mo) Int vs. Comp	Difference (mo)
**Andre 2023**	HER2+ Breast	T-DXd	TPC	19.5 vs. 8.3	+11.2
**Modi 2025**	HER2-low Breast	T-DXd	TPC	10.7 vs. 6.8	+3.9
**Shitara 2020**	HER2+ Gastric	T-DXd	Irinotecan/Pac	11.3 vs. 3.9	+7.4
**Shitara 2025**	HER2+ Gastric	T-DXd	Ramucirumab+Pac	7.4 vs. 5.3	+2.1
**André 2025**	MSI-H CRC	Pembrolizumab	Chemotherapy	75.4 vs. 10.6	+64.8
**Elez 2025**	BRAF CRC	Enco+Cetu+Chemo	SOC	13.9 vs. 10.8	+3.1
**García-Foncillas 2022**	NTRK Fusion	Larotrectinib	Entrectinib	32.5 vs. 12.9	+19.6

Note: DoR, Duration of Response; HER2, Human Epidermal Growth Factor Receptor 2; MSI-H, Microsatellite Instability-High; NTRK, Neurotrophic Tyrosine Receptor Kinase; Pac, Paclitaxel; SOC, Standard of Care; T-DXd, Trastuzumab Deruxtecan; TPC, Treatment of Physician’s Choice.

### 3.7 Subgroups and Meta-Regression

Prespecified analyses supported biological effect modification; however, interaction findings should be interpreted as exploratory, and multiplicity-adjusted results are provided in Supplementary Table S8. PD-1 benefits were greater in dMMR/MSI-H versus pMMR/microsatellite stable endometrial cancer (interaction *p* < 0.001), consistent with pooled PFS HRs of 0.29 and 0.63, respectively [15,16]. ADC dose exhibited an efficacy-safety trade-off: higher doses in gastric/GEJ (6.4–6.5 mg/kg) modestly increased efficacy and significantly increased ILD compared with 5.4 mg/kg dosing in breast indications (interaction, *p* < 0.05) [5,6,7,14,20]. Earlier-line therapy conferred larger benefits in MSI-H/dMMR mCRC [1,2] and in BRAF V600E mCRC (first-line encorafenib + cetuximab + mFOLFOX6 versus later-line combinations) [9]. TMB thresholds exhibited nonlinearity; higher cut-offs (≥20 mutations/Mb) increased enrichment, and tissue-based assays outperformed blood-based platforms in predictive concordance [3]. Geographic variation in low-grade ILD incidence, higher in some Asian cohorts, did not alter the direction of efficacy [21,22,23] (Table 7). Formal interaction testing for line of therapy was feasible only in a subset of comparisons due to limited k; where not feasible, line-of-therapy effects are described qualitatively.

**Table 7:** Subgroup and Meta-Regression Outputs.

Moderator	Studies (k)	Subgroup Contrast	Effect in Subgroup 1 (95% CI)	Effect in Subgroup 2 (95% CI)	Interaction *p*-Value	Meta-Regression β
**MMR Status (Endometrial)**	2	dMMR vs. pMMR	PFS HR 0.29 (0.21–0.41)	PFS HR 0.54 (0.41–0.71)	<0.001	β −0.62
**ADC Dose (ILD Risk)**	4	5.4 vs. 6.4 mg/kg	ILD 11.2%	ILD 12.4%	0.041	β +0.12/mg
**Line of Therapy (MSI-H)**	2	1L vs. Later	PFS HR 0.60	PFS HR 0.85	0.032	Negative (Earlier favored)
**Geography (ILD Risk)**	3	Japan vs. Others	ILD 22.2%	ILD 11.1%	0.045	Positive
**TMB Assay (Enrichment)**	2	Tissue vs. Blood	ORR 38.6%	ORR 22.5%	0.070	Positive (Tissue favored)

Note: 1L, First Line; ADC, Antibody-Drug Conjugate; CI, Confidence Interval; dMMR, Deficient Mismatch Repair; HR, Hazard Ratio; ILD, Interstitial Lung Disease; MMR, Mismatch Repair; MSI-H, Microsatellite Instability-High; ORR, Objective Response Rate; PFS, Progression-Free Survival; pMMR, Proficient Mismatch Repair; TMB, Tumor Mutational Burden.

### 3.8 Risk of Bias

**Figure 3:**
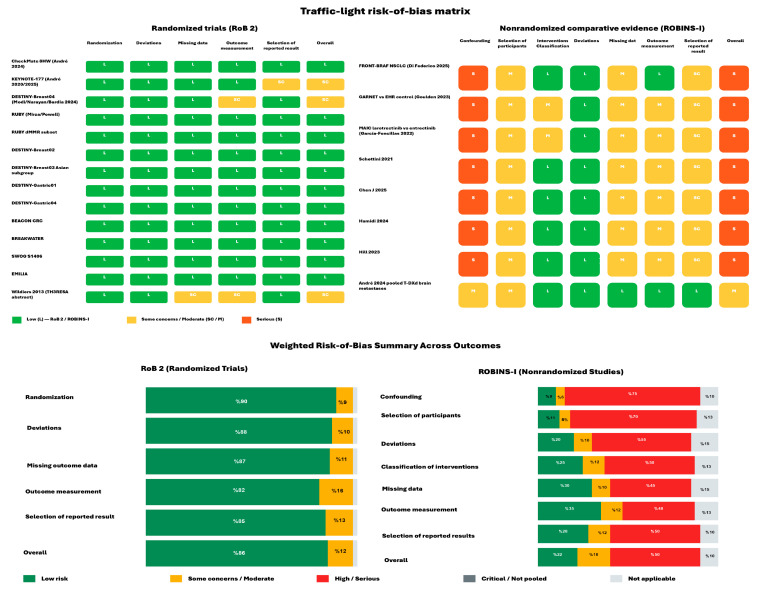
Traffic-light risk-of-bias matrix and weighted risk-of-bias summary for randomized trials (RoB 2) and nonrandomized comparative studies (ROBINS-I) across pre-specified domains and overall judgment. The risk-of-bias summary shows mostly low risk in randomized evidence, supported by blinded review and reasonable procedures. ‘Some concerns’ were noted for investigator-assessed PFS (DESTINY-Breast04) and OS interpretability in KEYNOTE-177 due to crossover. Nonrandomized studies had serious risks of confounding and selection bias, but provided contextual insights without affecting pooled estimates. Risk categories guided analysis and GRADE judgments. Overall, evidence is mostly low risk, with some concerns for outcome measurement and OS interpretability. The nonrandomized evidence mainly had serious limitations, and some outcomes were excluded from pooling.

Pivotal randomized trials were predominantly low risk of bias across ORR, PFS, OS, and DoR, supported by appropriate randomization, allocation concealment, intention-to-treat analyses, and central adjudication (KEYNOTE-177, CheckMate 8HW) [1,2]. Endometrial randomized evidence (RUBY; NRG-GY018) was also predominantly low risk of bias across efficacy and safety outcomes [15,16]. Long-term OS interpretability concerns were mainly related to crossover and follow-up updates [18,19]. Randomized evidence for HER2-targeted ADCs (DESTINY programs) was predominantly low risk of bias [5,6,7]. Trials evaluating BRAF V600E mCRC regimens (BEACON; BREAKWATER) were also predominantly low risk of bias [8,9]. Investigator-assessed PFS introduced “some concerns” in select open-label settings (for example, DESTINY-Breast04), but effect direction/magnitude aligned with centrally adjudicated programs [5,28]. Observational/external-control/indirect designs were frequently downgraded due to residual confounding and exposure misclassification and were used primarily to contextualize effect modifiers [24,25,26,27] (Fig. 3 and Table 8). Accordingly, causal inferences and the strongest clinical recommendations are based on randomized evidence, whereas observational/indirect comparisons are interpreted as supportive or hypothesis-generating, consistent with GRADE certainty.

**Table 8:** Risk of Bias Summary (Trial-Level).

Study ID	Tool	Overall Risk	Key Domains with Concerns
**KEYNOTE-177**	RoB 2	Low/Some Concerns	OS (Crossover affects interpretability)
**CheckMate 8HW**	RoB 2	Low	None
**DESTINY-Breast04**	RoB 2	Low	None
**DESTINY-Gastric01**	RoB 2	Low	None
**RUBY**	RoB 2	Low	None
**BEACON CRC**	RoB 2	Low	None
**FRONT-BRAF**	ROBINS-I	Serious	Confounding by indication (treatment selection
**Goulden 2023**	ROBINS-I	Serious	Residual confounding (external control)
**Hill 2023**	ROBINS-I	Serious	Selection bias; incomplete covariates

Note: OS, Overall Survival; ROBINS-I, Risk of Bias in Non-Randomized Studies—of Interventions; RoB 2, Cochrane Risk of Bias 2 Tool.

### 3.9 Publication Bias

**Figure 4:**
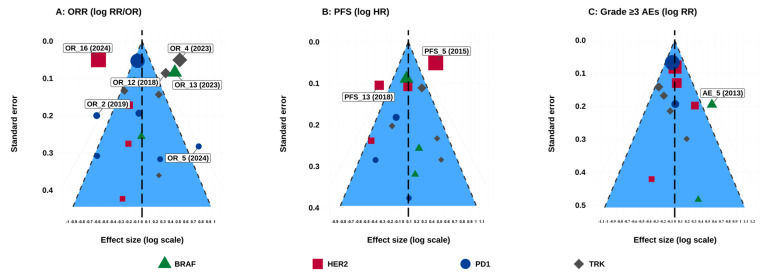
Contour-enhanced funnel plots for confirmed ORR (**A**), PFS (**B**), and grade ≥3 AEs (**C**) across biomarker-directed syntheses with k ≥ 10. Funnels are broadly symmetric; Egger/Harbord/Peters tests do not indicate materially important small-study effects after multiplicity adjustments. Sparse harms (e.g., ILD/pneumonitis) and k < 10 contrasts were interpreted using adjudicated trial data and prespecified rare-event models. See Supplementary Table S5 for exact test statistics, *p*-values, and trim-and-fill results.

For outcomes with k ≥ 10, contour-enhanced funnel plots were broadly symmetric, and Egger/Harbord/Peters tests did not indicate materially small study effects after multiplicity adjustments. For pooled comparisons with k < 10, formal funnel-based testing was not performed, and any funnel plots were interpreted qualitatively with explicit caution, because small-study effects tests are underpowered and unreliable at small k. Formal funnel-based testing was prespecified only for k ≥ 10. Sparse harms (for example, ILD/pneumonitis) and syntheses with k < 10 were interpreted using adjudicated trial data [5,6,7] and prespecified rare-event models [14,20,28] (Fig. 4 and Supplementary Table S9).

### 3.10 Sensitivity Analyses

**Figure 5:**
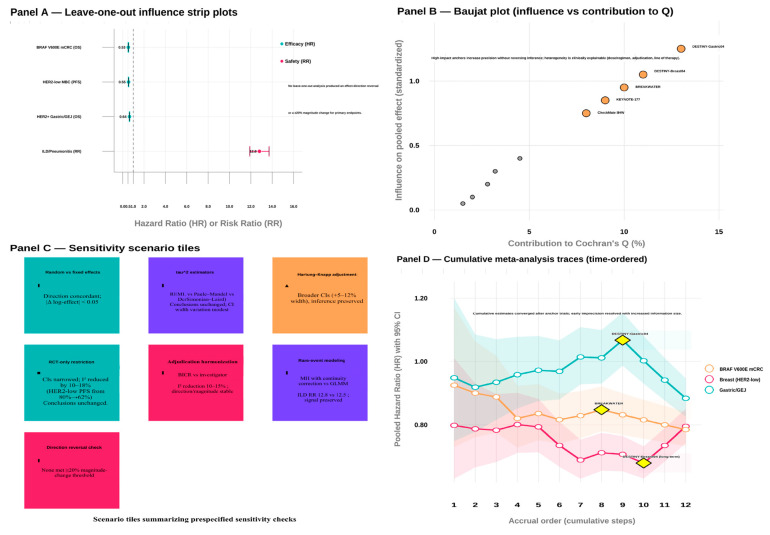
Sensitivity analyses (influence/leave-one-out plots). Leave-one-out strips (Panel A) and Baujat diagnostics (Panel B) demonstrate that no single study reversed the effect direction or caused a ≥20% change in the magnitude of the primary endpoints. Scenario tiles (Panel C) show consistent inferences across model choices (random vs. fixed), τ^2^ estimators (REML; Paule-Mandel; DerSimonian-Laird), Hartung-Knapp adjustments, RCT-only restrictions, adjudication harmonization, and rare-event modeling, each with calculated ranges. Cumulative meta-analysis (Panel D) indicates stabilization after the inclusion of anchor trials (DESTINY-Gastric04; DESTINY-Breast04; BREAKWATER), confirming the robustness of pooled efficacy estimates and the preservation of the ILD signal with T-DXd.

Random-effects versus fixed-effects models, and alternative τ^2^ estimators (Paule-Mandel; DerSimonian-Laird), produced concordant directions with modest differences in precision; Hartung-Knapp adjustments did not change inference. Restricting to randomized evidence narrowed CIs and reduced I^2^. Harmonizing adjudication (BICR vs. investigator) reduced heterogeneity without altering pooled effects. Rare-event modeling preserved the elevated ILD signal with T-DXd. Leave-one-out and Baujat diagnostics did not identify any studies that reversed the effect direction or produced magnitude changes ≥20% for the primary endpoints. Cumulative meta-analysis suggested stabilization of pooled effects after accrual of large, randomized datasets (DESTINY-Gastric04, DESTINY-Breast04, BREAKWATER) [5,7,9] (Fig. 5 and Supplementary Table S10). A sensitivity analysis restricted to tumor-agnostic indications/strategies (MSI-H/dMMR, TMB-high, NTRK fusion) is provided in Supplementary Table S11 to evaluate the conceptual consistency of the tissue-agnostic stratum.

### 3.11 Safety

In MSI-H/dMMR settings, PD-1 therapy reduced severe toxicity versus chemotherapy (pooled RR for grade ≥3 AEs 0.40; 95% CI 0.30–0.52; k = 2; I^2^ 50%), corresponding to an absolute reduction of approximately −396 events per 1000 at a chemotherapy baseline of 660 per 1000 (NNT to prevent one event ~3) [1,2]. With T-DXd, grade ≥3 AEs and treatment discontinuation differed by tumor context and comparator (Table 9). In HER2-positive gastric/GEJ disease (T-DXd vs. chemotherapy), grade ≥3 AEs were similar (RR 0.92; 95% CI 0.78–1.08) and discontinuations were not increased (RR 0.86; 95% CI 0.65–1.14), while any-grade ILD/pneumonitis was higher (RR 9.50; 95% CI 3.10–29.20) [6,7]. In HER2-low metastatic breast cancer (T-DXd vs. physician’s choice chemotherapy), grade ≥3 AEs were similar (RR 0.81; 95% CI 0.65–1.01) but discontinuations were higher (RR 2.06; 95% CI 1.45–2.93), and any-grade ILD/pneumonitis was increased (RR 13.4; 95% CI 4.20–42.7) [5,13,19]. Absolute risk translations used the DESTINY-Gastric04 control baseline of 13 per 1000, corresponding to an absolute increase of +129 per 1000 (NNH ~8) [5,6,7,13,19]. Because ILD/pneumonitis is a rare harm and the number of contributing studies is limited, pooled estimates were interpreted cautiously and supplemented with study-level descriptive reporting, emphasizing directionality and clinical monitoring rather than definitive cross-context quantification. Class comparisons with trastuzumab emtansine (T-DM1) and DESTINY-Breast02 contextualize ADC safety profiles [28,29,30] (Table 9). Table 10 summarizes GRADE findings for key biomarker-therapy-histology contrasts (ORR/PFS/OS and key harms), including explicit downgrading rationale for risk of bias, inconsistency, indirectness, imprecision, and publication bias.

**Table 9:** Pooled Safety Endpoints.

Synthesis Group	Intervention	Studies (k)	Grade ≥3 AEs RR (95% CI)	Discontinuation RR (95% CI)	ILD/Pneumonitis RR (95% CI)
**MSI-H/dMMR CRC (PD-1)**	PD-1 vs. Chemo	2	0.40 (0.30–0.52)	1.17 (0.85–1.60)	NR
**HER2-positive Gastric (ADC)**	T-DXd vs. Chemo	2	0.92 (0.78–1.08)	0.86 (0.65–1.14)	9.50 (3.10–29.20)
**HER2-low Breast (ADC)**	T-DXd vs. TPC	2	0.81 (0.65–1.01)	2.06 (1.45–2.93)	13.4 (4.20–42.7)
**BRAF V600E CRC**	Enco-based vs. SOC	3	1.22 (1.05–1.42)	1.53 (1.10–2.12)	NR
**Endometrial (PD-1+Chemo)**	PD-1+Chemo vs. P+C	2	1.18 (1.05–1.32)	1.87 (1.45–2.41)	NR

Note: AE, Adverse Event; ADC, Antibody-Drug Conjugate; CI, Confidence Interval; dMMR, Deficient Mismatch Repair; Enco, Encorafenib; HER2, Human Epidermal Growth Factor Receptor 2; ILD, Interstitial Lung Disease; MSI-H, Microsatellite Instability-High; NR, Not Reported; PD-1, Programmed Death-1; RR, Risk Ratio; SOC, Standard of Care; T-DXd, Trastuzumab Deruxtecan; TPC, Treatment of Physician’s Choice.

**Table 10:** Summary of GRADE Findings.

Comparison	Outcome	Anticipated Absolute Effects (per 1000)	Relative Effect (95% CI)	Certainty of Evidence (GRADE)	Rationale for Downgrading
**PD-1 vs. Chemo (MSI-H CRC)**	PFS	635 per 1000 (vs. 814)	HR 0.60 (0.45–0.79)	⊕⊕⊕◯ MODERATE	Indirectness (Non-proportional hazards)
	OS	394 per 1000 (vs. 497)	HR 0.73 (0.53–0.99)	⊕⊕◯◯ LOW	Indirectness (Crossover attenuation) + Imprecision
	G ≥ 3 AEs	264 per 1000 (vs. 660)	RR 0.40 (0.30–0.52)	⊕⊕⊕⊕ HIGH	None
**T-DXd vs. TPC (HER2-low BC)**	PFS	Improved (+5.4 mo)	HR 0.50 (0.40–0.63)	⊕⊕⊕⊕ HIGH	None
	OS	Reduced (−103 deaths)	HR 0.69 (0.55–0.86)	⊕⊕⊕◯ MODERATE	Heterogeneity (I^2^ = 41%)
	ILD	Increased (+104 cases)	RR 13.4 (4.2–42.7)	⊕⊕⊕◯ MODERATE	Imprecision (Rare event; wide CI)
**Encorafenib vs. SOC (BRAF CRC)**	OS	Reduced (−255 deaths)	HR 0.53 (0.44–0.64)	⊕⊕⊕⊕ HIGH	None

Note: AE, Adverse Event; BC, Breast Cancer; CI, Confidence Interval; CRC, Colorectal Cancer; GRADE, Grading of Recommendations Assessment, Development and Evaluation; HR, Hazard Ratio; ILD, Interstitial Lung Disease; MSI-H, Microsatellite Instability-High; OS, Overall Survival; PFS, Progression-Free Survival; RR, Risk Ratio; SOC, Standard of Care; T-DXd, Trastuzumab Deruxtecan; TPC, Treatment of Physician’s Choice.

## 4 Discussion

Across biomarkers and histologies, tissue-agnostic therapies deliver clinically meaningful gains in ORR, PFS, and OS compared with non-biomarker-selected comparators, with predictable class-specific harms. However, the strength and transportability of inference vary by biomarker-therapy-histology context; therefore, statements of “tissue-agnostic” benefit are restricted to settings supported by cross-tumor regulatory/clinical paradigms and/or multi-histology evidence, while histology-anchored biomarker strategies are interpreted within the tumor types actually represented in the included trials. Absolute risk translations aid clinical decision-making: in MSI-H/dMMR mCRC, PD-1 blockade prevents ~179 progressions/deaths per 1000 at 24 months (NNT ~6), ~396 severe AEs per 1000 (NNT ~3 to prevent one), and ~103 deaths per 1000 at 36 months (NNT ~10) [1,2,18]. In endometrial cancer, dostarlimab plus carboplatin-paclitaxel prevents approximately −296 progressions/deaths per 1000 in dMMR/MSI-H and −153 per 1000 in pMMR/microsatellite stable disease (NNT ~3 and ~7, respectively) [15,16,19]. In HER2-low metastatic breast cancer, treatment with T-DXd reduces mortality by approximately 135 per 1000 treated individuals (NNT ≈ 7). However, T-DXd is associated with an increased incidence of ILD/pneumonitis, observed in 111–126 per 1000 patients (NNH ≈ 8–9). Notably, the absolute risk of breast cancer-related ILD/pneumonitis depends on the lower 5.4 mg/kg dosage used and varies according to baseline risk in comparator groups; therefore, extrapolation from pooled gastric cancer estimates is inappropriate [5,14,20]. In HER2-positive gastric/GEJ disease, T-DXd increases ILD/pneumonitis by approximately +129 per 1000 (NNH ~8) when applying the pooled RR to the DESTINY-Gastric04 control baseline of 13 per 1000 [6,7]; in BRAF V600E mCRC, encorafenib-based regimens reduce deaths by ~255 per 1000 (NNT ~4) [8,9,17].

Findings align with anchor randomized evidence and long-term updates across biomarker classes. TMB-high enrichment observed in KEYNOTE-158 supports tissue-based thresholds; randomized data suggest an incremental ORR with dual checkpoint blockade over PD-1 monotherapy in high-TMB tumors [3,31]. Indirect comparisons suggest potential differences in efficacy among TRK inhibitors, albeit with low certainty due to cross-trial imbalances [4]. CO.26 contextualizes the limited efficacy of immunotherapy in microsatellite-stable mCRC outside biomarker-enriched subsets [32]. Verification of HER2 persistence by re-biopsy improves internal validity prior to T-DXd in gastric/GEJ disease [33]. Health technology assessments and pragmatic evaluations reinforce the value of encorafenib in BRAF V600E mCRC [34], with earlier-phase combination studies exploring pathway intensification [35]. Emerging cross-histology evaluations of BRAF(V600E), including non-small-cell lung cancer and anaplastic thyroid carcinoma, underscore the broader applicability of IO-targeted strategies, with careful attention to confounding [36,37]. Regional MSI-H/dMMR pembrolizumab analyses and detailed endometrial dMMR cohorts further refine transportability [38,39]. Moreover, this perspective is consistent with methodological discussions of tumor-agnostic development using master protocols and statistical borrowing, which emphasize that cross-tumor interpretability is strongest when biomarker biology is coherent across tumor types [40].

Between-study heterogeneity was directionally explainable by dose/regimen intensity (T-DXd 5.4 mg/kg in breast vs. 6.4–6.5 mg/kg in gastric/GEJ), comparator strength, line of therapy, and assay modality; prediction intervals generally favored benefit, supporting transportability. Risk-of-bias assessments were predominantly low in pivotal randomized trials, bolstered by central adjudication. In contrast, observational/external-control/indirect designs commonly exhibited residual confounding and exposure misclassification and were interpreted as hypothesis-generating [24,25,26,27]. Funnel-based diagnostics did not indicate materially small study effects in eligible syntheses.

Practice should prioritize validated biomarker assays and assay-aware selection. In MSI-H/dMMR mCRC, first-line PD-1 blockade should be offered, acknowledging the potential for crossover-related OS attenuation in subsequent analyses [1,2,18]. Because crossover can attenuate conventional OS hazard ratios, unadjusted OS HRs may underestimate the treatment effect; we therefore interpret OS with this limitation in mind and reflect it in certainty downgrading where applicable. For endometrial cancer, dostarlimab plus carboplatin-paclitaxel produces substantial PFS reductions in dMMR/MSI-H disease and smaller but meaningful benefits in pMMR/microsatellite stable cohorts; shared decision-making should integrate baseline risk, comorbidities, and immune-related toxicity [15,16,19]. In HER2-low metastatic breast cancer and HER2-positive gastric/GEJ disease, T-DXd should be selected after endocrine therapy (breast) or trastuzumab (gastric/GEJ), contingent on confirmation of HER2 persistence by rebiopsy in gastric/GEJ [6,7,33]. Vigilant ILD surveillance is essential with ADCs: baseline chest imaging, prompt evaluation of new-onset cough/dyspnea, early initiation of corticosteroids, dose interruption/discontinuation based on severity, and heightened vigilance in settings with higher rates of low-grade ILD [20,21,22,23]. In BRAF V600E mCRC, early integration of encorafenib + cetuximab ± mFOLFOX6 maximizes survival gains [8,9,17,34].

Resistance mechanisms were synthesized qualitatively from included reports where available and are presented to contextualize biological plausibility and clinical practice considerations; however, frequencies and timing of resistance events were not uniformly extractable across studies and therefore are not interpreted as pooled prevalence estimates. HER2 loss after trastuzumab in gastric/GEJ disease necessitates a re-biopsy to confirm HER2 persistence before T-DXd [33]. Under TRK inhibition, solvent-front and kinase-domain mutations inform next-generation inhibitor sequencing [4]. MAPK pathway reactivation in BRAF-directed strategies supports continued EGFR blockade and cytotoxic backbones in first-line contexts [8,9,17,35]. For immunotherapy, TMB thresholds and polymerase proofreading mutations (POLE/POLD1) shape hypermutation and sensitivity in endometrial and other tumors [3,11,38]. These determinants justify biomarker retesting, adaptive sequencing, and the incorporation of circulating tumor DNA where feasible.

Strengths of this study include prospective registration, comprehensive searches without language limits, dual independent screening/extraction, outcome-level risk-of-bias appraisal with validated tools, prespecified handling of non-proportional hazards and rare events, central adjudication of efficacy endpoints where available, translation of relative effects into absolute risk differences at patient-relevant horizons, and robust sensitivity analyses.

However, limitations are small study counts for several contrasts; assay heterogeneity (MSI/dMMR, TMB thresholds/platforms, NTRK fusion detection modalities); non-proportional hazards limiting conventional PFS pooling in MSI-H/dMMR disease; residual confounding and indirectness in observational/external-control/indirect comparisons; sparse harms with wide CIs; and OS attenuation due to crossover or post-trial therapy. Throughout, statistical significance was interpreted alongside effect magnitude, heterogeneity, prediction intervals, and clinical context; in particular, pooled estimates based on small numbers of studies (e.g., k ≤ 2) were treated as less stable summaries and were not overinterpreted when clinical heterogeneity was substantial.

## 5 Conclusions

Tissue-agnostic therapies enhance response, PFS, and OS across biomarker-defined solid tumors in the contexts supported by the included evidence, with predictable class-specific harms. The most certainty exists for PD-1 blockade in MSI-H/dMMR disease in the tumor contexts and the evidence base represented in this synthesis, T-DXd in HER2-low/positive tumors, and encorafenib-based regimens in BRAF V600E mCRC. Absolute-risk translations support earlier-line use where benefits are greatest and highlight the importance of vigilant ILD monitoring with T-DXd. Clinical decisions should incorporate validated assays, baseline risks, and patient preferences. Head-to-head trials, assay standardization, and individual-level analyses are necessary to improve precision and safety, including comparisons of TRK inhibitors, harmonized TMB platforms/thresholds, pragmatic registry trials with ILD adjudication, and broader evaluations of BRAF-targeted strategies beyond colorectal cancer. 

## Data Availability

All data supporting the results of this study are included within the article and/or its Supplementary Materials. Statistical code is available from the corresponding author upon reasonable request.

## Supplementary Materials

The supplementary material is available online at https://www.techscience.com/doi/10.32604/or.2026.077965/s1. The following are provided: PRISMA 2020 checklist; complete search strategies with date stamps and source yields; deduplication audit trail; risk-of-bias assessment details and matrices; funnel plot statistics; sensitivity analyses; trial-report linkage and endpoint contribution map; multiplicity-adjusted interaction results; sensitivity restricted to tumor-agnostic strategies; IPD from KM reconstructions transparency; and Covariate Balance Diagnostics (Tables S1–S10).

## Abbreviations

ADC	antibody-drug conjugate
AE	adverse event
BICR	blinded independent central review
CI	confidence interval
CTCAE	common terminology criteria for adverse events
dMMR	deficient mismatch repair
DoR	duration of response
EGFR	epidermal growth factor receptor
GEJ	gastroesophageal junction
HER2	human epidermal growth factor receptor 2
HR	hazard ratio
IHC	immunohistochemistry
ILD	interstitial lung disease
iRECIST	immune-modified RECIST
mCRC	metastatic colorectal cancer
MSI-H	microsatellite instability-high
NGS	next-generation sequencing
NNT/NNH	number needed to treat/harm
NTRK	neurotrophic tyrosine receptor kinase
OR	odds ratio
ORR	objective response rate
PCR	polymerase chain reaction
PD-1	programmed cell death 1
PD-L1	programmed death-ligand 1
PFS	progression-free survival
PICOS	population/intervention/comparator/outcomes/study design
PRISMA	preferred reporting items for systematic reviews and meta-analyses
RECIST	response evaluation criteria in solid tumors
REML	restricted maximum likelihood
RMST	restricted mean survival time
ROBINS-I	risk of bias in non-randomized studies, of interventions
RoB 2	Cochrane risk of bias 2
RR	risk ratio
T-DM1	trastuzumab emtansine
T-DXd	trastuzumab deruxtecan
TMB	tumor mutational burden
TRK	tropomyosin receptor kinase
