# Supplementary material for: Tissue-Agnostic Targeting in Solid Tumors: A PRISMA-Compliant Meta-Analysis of Efficacy, Safety, and Resistance Determinants Across Histologies
Source: Oncol Res. 2026 Jun 16;34(7):5. doi: 10.32604/or.2026.077965 (PMC13292025; doi:10.32604/or.2026.077965)
Supplement: Supplementary file 1 [file OncolRes-34-77965-s001.zip › TSP_OR_77965-s001.docx]

- **PRISMA checklist:**

**‎**

| **Section and Topic** | **Item No** | **Checklist item** | **Reported on Page Number/Line Number** | **Reported on Section/Paragraph** |  |  |
| --- | --- | --- | --- | --- | --- | --- |
| **TITLE** | | |  |  |  |  |
| Title | 1 | Identify the report as a systematic review. | 1/3-5 | Title; Abstract (first paragraph: “PRISMA-compliant systematic review and random-effects meta-analysis”) |  |  |
| **ABSTRACT** | | |  |  |  |  |
| Abstract | 2 | See the PRISMA 2020 for Abstracts checklist. | 1/14-37 | Abstract (entire section: objectives, data sources, eligibility, methods, results, conclusions) |  |  |
| **INTRODUCTION** | | |  |  |  |  |
| Rationale | 3 | Describe the rationale for the review in the context of existing knowledge. | 2-3/50-79 | Introduction 1, paragraphs 1-2 (precision oncology context; biomarker classes; clinical implementation challenges) |  |  |
| Objectives | 4 | Provide an explicit statement of the objective(s) or question(s) the review addresses. | 2-4/48-97 | Introduction 1, final paragraph; Methods 2.2 (PICOS and prespecified clinical question) |  |  |
| **METHODS** | | |  |  |  |  |
| Eligibility criteria | 5 | Specify the inclusion and exclusion criteria for the review and how studies were grouped for the syntheses. | 4/102-121 | Methods 2.2 Eligibility criteria (biomarker-positive advanced/metastatic solid tumors; interventions; comparators; outcomes; designs) |  |  |
| Information sources | 6 | Specify all databases, registers, websites, organisations, reference lists and other sources searched or consulted to identify studies. Specify the date when each source was last searched or consulted. | 4-5/123-131 | Methods 2.3 Information sources and search strategy (databases, trial registries, grey literature; PRESS revision dated June 10, 2025; Supplementary Materials and Table S1) |  |  |
| Search strategy | 7 | Present the full search strategies for all databases, registers and websites, including any filters and limits used. | ‎‎4-5/123-131‎ | Methods 2.3; Supplementary Materials (full strategies, date stamps, de-duplication details) |  |  |
| Selection process | 8 | Specify the methods used to decide whether a study met the inclusion criteria of the review, including how many reviewers screened each record and each report retrieved, whether they worked independently, and if applicable, details of automation tools used in the process. | 5/133-138 | Methods 2.4 Study selection (dual independent screening; consensus resolution; adjudication; overlap handling; cluster/multi-arm adjustments) |  |  |
| Data collection process | 9 | Specify the methods used to collect data from reports, including how many reviewers collected data from each report, whether they worked independently, any processes for obtaining or confirming data from study investigators, and if applicable, details of automation tools used in the process. | 5/140-150 | Methods 2.5 Data extraction and outcome harmonization (dual extraction; template; data dictionary; adjudication; reconstruction from KM where needed) |  |  |
| Data items | 10a | List and define all outcomes for which data were sought. Specify whether all results that were compatible with each outcome domain in each study were sought (e.g. for all measures, time points, analyses), and if not, the methods used to decide which results to collect. | ‎‎5/140-150‎ | Methods 2.5 and Table 2 (outcomes: ORR, PFS, OS, DoR, grade ≥3 AEs, discontinuations, ILD/pneumonitis, irAEs, molecular resistance; adjudication hierarchy) |  |  |
|  | 10b | List and define all other variables for which data were sought (e.g. participant and intervention characteristics, funding sources). Describe any assumptions made about any missing or unclear information. | ‎‎5/140-150‎ | Methods 2.5 (study IDs, design, setting, populations, follow-up, funding/conflicts, intervention/comparator details, covariates; assumptions and flags for reconstructed HRs) |  |  |
| Study risk of bias assessment | 11 | Specify the methods used to assess risk of bias in the included studies, including details of the tool(s) used, how many reviewers assessed each study and whether they worked independently, and if applicable, details of automation tools used in the process. | 5/152-156 | Methods 2.6 Risk of bias (RoB 2 for RCTs; ROBINS-I for nonrandomized; target trial emulation; outcome-level assessment; dual reviewer approach; Supplementary matrices) |  |  |
| Effect measures | 12 | Specify for each outcome the effect measure(s) (e.g. risk ratio, mean difference) used in the synthesis or presentation of results. | 5-6/158-178 | Methods 2.7 Statistical analysis (ORR/AEs/discontinuations as RR; time-to-event as HR; RMST differences; absolute risk translations; NNT/NNH) |  |  |
| Synthesis methods | 13a | Describe the processes used to decide which studies were eligible for each synthesis (e.g. tabulating the study intervention characteristics and comparing against the planned groups for each synthesis (item #5)). | ‎5-6/133-178‎ | Methods 2.5 and 2.7 (eligibility mapping to biomarker-treatment syntheses; central adjudication prioritization; handling of multi-arm/cluster trials per Cochrane guidance) |  |  |
|  | 13b | Describe any methods required to prepare the data for presentation or synthesis, such as handling of missing summary statistics, or data conversions. | 5-6/140-178 | Methods 2.5 and 2.7 (KM-derived HR reconstruction; continuity corrections; GLMM/beta-binomial for sparse harms; OR-to-RR conversions) |  |  |
|  | 13c | Describe any methods used to tabulate or visually display results of individual studies and syntheses. | ‎‎5-6/158-178‎ | Methods 2.7; Results 3.2-3.6 (structured tables 1-6; forest plots described in Figure 2; funnel plots Figure 4; influence plots Figure 5) |  |  |
|  | 13d | Describe any methods used to synthesize results and provide a rationale for the choice(s). If meta-analysis was performed, describe the model(s), method(s) to identify the presence and extent of statistical heterogeneity, and software package(s) used. | ‎‎5-6/158-178‎ | Methods 2.7 (random-effects REML; fixed-effect sensitivity; τ² estimators; Hartung-Knapp; heterogeneity metrics Q/I²/τ²; prediction intervals; software: R [metafor, meta, robumeta, clubSandwich, survival, IPDfromKM, glmmTMB/lme4, dmetar], Jamovi) |  |  |
|  | 13e | Describe any methods used to explore possible causes of heterogeneity among study results (e.g. subgroup analysis, meta-regression). | ‎‎5-6/158-178‎ | Methods 2.7 (prespecified subgroup/meta-regression moderators including dose, line, comparator, assay modality, PD-L1, ECOG, age, geography; restricted cubic splines) |  |  |
|  | 13f | Describe any sensitivity analyses conducted to assess robustness of the synthesized results. | ‎‎5-6/158-178‎ | Methods 2.7 (model choices, adjudication harmonization, RCT-only restrictions, rare-event modeling, leave-one-out/Baujat) |  |  |
| Reporting bias assessment | 14 | Describe any methods used to assess risk of bias due to missing results in a synthesis (arising from reporting biases). | ‎‎5-6/158-178‎ | Methods 2.7 (contour-enhanced funnels; Egger/Harbord/Peters; trim-and-fill; selection models ‎exploratory); Supplementary Methods (Publication bias testing: exact ‎tests, effect-size metrics, software/functions, and k-threshold ‎eligibility) ‎ |  |  |
| Certainty assessment | 15 | Describe any methods used to assess certainty (or confidence) in the body of evidence for an outcome. | ‎‎5-6/158-178‎ | Methods 2.7 (GRADE framework; translation to absolute effects and NNT/NNH; certainty judgments informed by RoB and heterogeneity) |  |  |
| **RESULTS** | | |  |  |  |  |
| Study selection | 16a | Describe the results of the search and selection process, from the number of records identified in the search to the number of studies included in the review, ideally using a flow diagram. | 6/182-186 | Results 3.1 Study selection; Figure 1 PRISMA flow diagram (records identified, duplicates removed, screened, excluded with reasons, included) |  |  |
|  | 16b | Cite studies that might appear to meet the inclusion criteria, but which were excluded, and explain why they were excluded. | ‎‎6/182-186‎ | Results 3.1; Figure 1 exclusion categories (e.g., grey literature/no extractable outcomes; nonclinical; secondary research; insufficient design/analysis; diagnostic-only; ineligible biomarker/therapy); Supplementary Table S2 |  |  |
| Study characteristics | 17 | Cite each included study and present its characteristics. | 6-13/188-244 | Results 3.2 Study characteristics; Tables 1-2; Supplementary Table S6 (design, setting, interventions, comparators, endpoints) |  |  |
| Risk of bias in studies | 18 | Present assessments of risk of bias for each included study. | 37-41/365-391 | Results 3.8 Risk of bias; Figure 3 traffic-light matrix and summary; Table 8 study-level RoB with justifications; Supplementary materials |  |  |
| Results of individual studies | 19 | For all outcomes, present, for each study: (a) summary statistics for each group (where appropriate) and (b) an effect estimate and its precision (e.g. confidence/credible interval), ideally using structured tables or plots. | 13-30/246-337 | Results 3.3-3.6; Tables 3-6 (ORR, PFS, OS, DoR per study with HR/RR and 95% CI); Figure 2 (forest plot narrative) |  |  |
| Results of syntheses | 20a | For each synthesis, briefly summarise the characteristics and risk of bias among contributing studies. | ‎‎‎13-41‎/246-391‎ | Results 3.2-3.6; 3.8 (mix of RCTs and nonrandomized; predominantly low risk in pivotal trials; observational limitations noted) |  |  |
|  | 20b | Present results of all statistical syntheses conducted. If meta-analysis was done, present for each the summary estimate and its precision (e.g. confidence/credible interval) and measures of statistical heterogeneity. If comparing groups, describe the direction of the effect. | ‎‎‎13-30/246-337‎ | Results 3.2-3.6 (pooled RRs/HRs with 95% CIs; heterogeneity I²/τ²/Q; RMST differences); 3.11 Safety (pooled RRs, NNT/NNH) |  |  |
|  | 20c | Present results of all investigations of possible causes of heterogeneity among study results. | 30-37/339-363 | Results 3.7 Subgroups and meta-regression (dose, line, assay modality, geography, TMB thresholds; interaction p-values; β directions) |  |  |
|  | 20d | Present results of all sensitivity analyses conducted to assess the robustness of the synthesized results. | 41-42/394-413 | Results 3.10 Sensitivity analyses; Figure 5; Supplementary Table S9 (influence, leave-one-out, scenario analyses) |  |  |
| Reporting biases | 21 | Present assessments of risk of bias due to missing results (arising from reporting biases) for each synthesis assessed. | 41/381-392 | Results 3.9 Publication bias; Figure 4; Supplementary Table S8 (funnel tests; trim-and-fill); ‎explicit caution that formal funnel-based testing is unreliable ‎when k<10‎ |  |  |
| Certainty of evidence | 22 | Present assessments of certainty (or confidence) in the body of evidence for each outcome assessed. | ‎‎‎5-6/158-178‎‎  ‎‎13-30/246-337‎‎‎ | Methods 2.7 (GRADE planned); Results 3.2-3.6 (interpretation aligned with trial quality and heterogeneity); Discussion 4; (certainty statements); Table 10 (Summary of GRADE ‎Findings) ‎ |  |  |
| **DISCUSSION** | | |  |  |  |  |
| Discussion | 23a | Provide a general interpretation of the results in the context of other evidence. | 46-48/436-509 | Discussion 4, paragraphs 1-3 (alignment with anchor trials; absolute risk translations; practice implications) |  |  |
|  | 23b | Discuss any limitations of the evidence included in the review. | ‎‎46-48/436-509‎‎ | Discussion 4, “Strengths and limitations” (small study counts; assay heterogeneity; non-proportional hazards; residual confounding; sparse harms; crossover effects) |  |  |
|  | 23c | Discuss any limitations of the review processes used. | ‎‎‎‎46-48/436-509‎ | Discussion 4 (observational/external-control/indirect design constraints; indirectness; measurement variability) |  |  |
|  | 23d | Discuss implications of the results for practice, policy, and future research. | ‎‎46-48/436-509‎ | Discussion 4; Conclusions 5 (assay validation; earlier-line use; ILD monitoring; need for head-to-head trials; TMB standardization; pragmatic registry trials) |  |  |
| **OTHER INFORMATION** | | |  |  |  |  |
| Registration and protocol | 24a | Provide registration information for the review, including register name and registration number, or state that the review was not registered. | ‎‎4/95-121‎ | Methods 2.1 Protocol and registration (PROSPERO CRD420251235402) |  |  |
|  | 24b | Indicate where the review protocol can be accessed, or state that a protocol was not prepared. | ‎‎4/102-121‎ | Methods 2.1; Supplementary Materials (registered protocol details; prespecified analyses) |  |  |
|  | 24c | Describe and explain any amendments to information provided at registration or in the protocol. | ‎‎4-5/102-131‎ | Methods 2.3 (PRESS-revised search strategy June 10, 2025, refining HER2-low and TRK terms; noted in Supplementary Table S1) |  |  |
| Support | 25 | Describe sources of financial or non-financial support for the review, and the role of the funders or sponsors in the review. | 48/522-‎543‎ | Statements 6 Funding Statement (PSAU/2025/03/25235; no role in design, analysis, or writing) |  |  |
| Competing interests | 26 | Declare any competing interests of review authors. | 48/522-‎543‎ | Statements 6 Conflicts of Interest (authors declare no conflicts) |  |  |
| Availability of data, code and other materials | 27 | Report which of the following are publicly available and where they can be found: template data collection forms; data extracted from included studies; data used for all analyses; analytic code; any other materials used in the review. | 48/522-‎543‎ | Statements 6 Availability of Data and Materials (all data within article/Supplementary; statistical code available from corresponding author upon request; Supplementary includes PRISMA checklist, search strategies, deduplication audit trail, risk-of-bias matrices, funnel statistics, sensitivity analyses) |  |  |

As the checklist was provided upon initial submission, the page number/line number reported may be changed due to copyediting and may not be referable in the published version. In this case, the section/paragraph may be used as an alternative reference.

**Supplementary Methods: Information Sources, Search Strategies, Date Stamps, and Deduplication**

**1. Sources of information and coverage periods**

The search included various bibliographic databases, such as MEDLINE via PubMed (2000 to the search date); Cochrane CENTRAL (from inception to present); Web of Science Core Collection and Scopus (from 2000 onward); and OpenAlex (January 1, 2000, to the search date). It also covered clinical trial registries like ClinicalTrials.gov, the EU Clinical Trials Register (EudraCT/EU CTR), and WHO ICTRP. Additionally, conference proceedings and society publications, such as the ASCO Annual Meeting, ESMO Congress, and AACR Annual Meeting, were reviewed. To find grey literature, preprint servers including medRxiv, bioRxiv, ProQuest, Google Scholar, CrossRef, Semantic Scholar, and LENS.org were examined. The inclusion period ranged from January 1, 2000, through the date of the last comprehensive search; earlier studies were included only if their biomarker definitions and therapeutic agents matched current standards.

**2. Search strategy development and peer review (PRESS)**

The strategy construction involved a clinician-information specialist team developing multi-concept queries that integrated biomarker terms, therapeutic agents, disease contexts, and comparative study filters. Synonym expansion and ontology terms were employed to enhance recall. An experienced health sciences information specialist peer-reviewed the entire strategies on June 10, 2025. Revisions made during the review included adding HER2-low terminology and ERBB2 synonyms, incorporating next-generation TRK inhibitors such as selitrectinib and repotrectinib, expanding tumor mutational burden synonyms, such as hypermutated, and correcting field tags. Additionally, proximity operators were refined for greater precision, particularly in Web of Science and Scopus databases.

**3. Database-specific strategies**

The structures below illustrate Boolean logic and concept blocks used across sources. Searches covered January 1, 2000, through October 21, 2025. No language restrictions were applied during the search; human subjects filters were used where appropriate.

**- MEDLINE (via PubMed)**

- Platform: PubMed (MEDLINE)

- Coverage window: 2000-01-01 to 2025-10-21

- Filters/limits applied at search: Humans; publication date from 2000/01/01 to 2025/10/21; no language restriction

- Verbatim search string:

("Neoplasms"[Mesh] OR neoplasm[tiab] OR tumor[tiab] OR carcinoma[tiab] OR "solid tumor"[tiab]) AND ( ("Microsatellite Instability"[Mesh] OR "DNA Mismatch Repair"[Mesh] OR microsatellite instab[tiab] OR MSI-H[tiab] OR dMMR[tiab] OR "mismatch repair deficien"[tiab]) OR ("Tumor Mutational Burden"[tiab] OR TMB[tiab] OR hypermutat[tiab] OR "mutational burden"[tiab]) OR (NTRK1[tiab] OR NTRK2[tiab] OR NTRK3[tiab] OR "neurotrophic tyrosine receptor kinase"[tiab] OR "TRK fusion"[tiab] OR ("Gene Fusion"[Mesh] AND (TRK[tiab] OR NTRK[tiab]))) OR ("Proto-Oncogene Proteins B-raf"[Mesh] OR BRAF[tiab] OR "BRAF V600E"[tiab] OR "BRAF V600K"[tiab] OR "BRAF V600R"[tiab] OR "BRAF V600D"[tiab]) OR ("Receptor, ErbB-2"[Mesh] OR HER2[tiab] OR ERBB2[tiab] OR "HER2-low"[tiab] OR "IHC 1+"[tiab] OR "IHC 2+"[tiab]) ) AND ( pembrolizumab[tiab] OR dostarlimab[tiab] OR nivolumab[tiab] OR ipilimumab[tiab] OR larotrectinib[tiab] OR entrectinib[tiab] OR selitrectinib[tiab] OR repotrectinib[tiab] OR encorafenib[tiab] OR binimetinib[tiab] OR cetuximab[tiab] OR dabrafenib[tiab] OR trametinib[tiab] OR vemurafenib[tiab] OR "trastuzumab deruxtecan"[tiab] OR T-DXd[tiab] OR "trastuzumab emtansine"[tiab] OR T-DM1[tiab] OR "antibody-drug conjugate"[tiab] ) AND ( randomized[tiab] OR randomised[tiab] OR trial[tiab] OR "phase 2"[tiab] OR "phase II"[tiab] OR "phase 3"[tiab] OR "phase III"[tiab] OR cohort[tiab] OR "case-control"[tiab] OR observational[tiab] OR comparative[tiab] OR controlled[tiab] )

**- Cochrane CENTRAL**

- Platform: Cochrane Library (CENTRAL)

- Coverage window: Inception to 2025-10-21

- Filters/limits: Trials only (inherent to CENTRAL); no language limits

- Verbatim search string (Advanced Search):

(Neoplasm OR tumor OR carcinoma OR "solid tumor") AND ( "microsatellite instab" OR MSI-H OR dMMR OR "mismatch repair deficien" OR "tumor mutational burden" OR TMB OR hypermutat OR NTRK1 OR NTRK2 OR NTRK3 OR "neurotrophic tyrosine receptor kinase" OR "TRK fusion" OR BRAF OR "BRAF V600E" OR "BRAF V600K" OR "BRAF V600R" OR "BRAF V600D" OR HER2 OR ERBB2 OR "HER2-low" OR "IHC 1+" OR "IHC 2+" ) AND ( pembrolizumab OR dostarlimab OR nivolumab OR ipilimumab OR larotrectinib OR entrectinib OR selitrectinib OR repotrectinib OR encorafenib OR binimetinib OR cetuximab OR dabrafenib OR trametinib OR vemurafenib OR "trastuzumab deruxtecan" OR T-DXd OR "trastuzumab emtansine" OR T-DM1 OR "antibody-drug conjugate" )

**- Web of Science Core Collection**

- Platform/Indexes: SCI-EXPANDED; SSCI; ESCI

- Coverage window: 2000-01-01 to 2025-10-20

- Filters/limits: Document types = Article OR Article in Press OR Proceedings Paper; Year ≥2000; no language limits

- Verbatim search string (Advanced Search; Topic = TS; proximity NEAR/x):

TS=( (neoplasm OR tumor OR carcinoma OR "solid tumor") AND ( ("microsatellite instab" OR MSI-H OR dMMR OR "mismatch repair deficien") OR ("tumor mutational burden" OR TMB OR hypermutat) OR (NTRK1 OR NTRK2 OR NTRK3 OR "neurotrophic tyrosine receptor kinase" OR "TRK fusion") OR (BRAF OR "BRAF V600E" OR "BRAF V600K" OR "BRAF V600R" OR "BRAF V600D") OR (HER2 OR ERBB2 OR "HER2-low" OR "IHC 1+" OR "IHC 2+") ) AND ( pembrolizumab OR dostarlimab OR nivolumab OR ipilimumab OR larotrectinib OR entrectinib OR selitrectinib OR repotrectinib OR encorafenib OR binimetinib OR cetuximab OR dabrafenib OR trametinib OR vemurafenib OR "trastuzumab deruxtecan" OR T-DXd OR "trastuzumab emtansine" OR T-DM1 OR "antibody-drug conjugate" ) AND ( random OR trial OR phase NEAR/2 (II OR III OR 2 OR 3) OR cohort OR "case-control" OR observational OR comparative OR controlled ) )

**- Scopus (Elsevier)**

- Platform: Scopus

- Coverage window: 2000-01-01 to 2025-10-20

- Filters/limits: Document type = Article OR Article-in-Press OR Conference Paper; Year ≥2000; no language limits

- Verbatim search string (TITLE-ABS-KEY):

TITLE-ABS-KEY( (neoplasm OR tumor OR carcinoma OR "solid tumor") AND ( ("microsatellite W/2 instab" OR MSI-H OR dMMR OR "mismatch W/2 repair W/2 deficien") OR ("tumor W/2 mutational W/2 burden" OR TMB OR hypermutat) OR (NTRK1 OR NTRK2 OR NTRK3 OR "neurotrophic W/2 tyrosine W/2 receptor W/2 kinase" OR "TRK W/2 fusion") OR (BRAF OR "BRAF W/1 V600E" OR "BRAF W/1 V600K" OR "BRAF W/1 V600R" OR "BRAF W/1 V600D") OR (HER2 OR ERBB2 OR "HER2-low" OR "IHC W/1 1+" OR "IHC W/1 2+") ) AND ( pembrolizumab OR dostarlimab OR nivolumab OR ipilimumab OR larotrectinib OR entrectinib OR selitrectinib OR repotrectinib OR encorafenib OR binimetinib OR cetuximab OR dabrafenib OR trametinib OR vemurafenib OR "trastuzumab W/1 deruxtecan" OR T-DXd OR "trastuzumab W/1 emtansine" OR T-DM1 OR "antibody-drug W/1 conjugate" ) AND ( random OR trial OR phase W/2 (II OR III OR 2 OR 3) OR cohort OR "case-control" OR observational OR comparative OR controlled ) )

**- OpenAlex**

- Platform: OpenAlex

- Coverage window: 2000-01-01 to 2025-10-20

- Filters: type = journal-article; human‎; no language limits

- Verbatim:

(neoplasm OR tumor OR carcinoma OR "solid tumor") AND ("microsatellite instab" OR MSI-H OR dMMR OR "mismatch repair deficien" OR "tumor mutational burden" OR TMB OR hypermutat OR NTRK1 OR NTRK2 OR NTRK3 OR "neurotrophic tyrosine receptor kinase" OR "TRK fusion" OR BRAF OR "BRAF V600E" OR "BRAF V600K" OR "BRAF V600R" OR "BRAF V600D" OR HER2 OR ERBB2 OR "HER2-low" OR "IHC 1+" OR "IHC 2+") AND (pembrolizumab OR dostarlimab OR nivolumab OR ipilimumab OR larotrectinib OR entrectinib OR selitrectinib OR repotrectinib OR encorafenib OR binimetinib OR cetuximab OR dabrafenib OR trametinib OR vemurafenib OR "trastuzumab deruxtecan" OR T-DXd OR "trastuzumab emtansine" OR T-DM1 OR "antibody-drug conjugate")

**- ClinicalTrials.gov**

- Platform: ClinicalTrials.gov Advanced Search

- Coverage window: All years to 2025-10-21

- Filters/limits: Study type = Interventional OR Observational; Recruitment = All; Results = All; Age = Child, Adult, Older Adult

- Verbatim query

Condition/Disease: neoplasm OR "solid tumor"

Other Terms:

( MSI-H OR dMMR OR "microsatellite instability" OR "mismatch repair deficiency" OR "tumor mutational burden" OR TMB OR hypermutat OR NTRK1 OR NTRK2 OR NTRK3 OR "TRK fusion" OR "BRAF V600E" OR "BRAF V600K" OR "BRAF V600R" OR "BRAF V600D" OR HER2 OR ERBB2 OR "HER2-low" ) AND ( pembrolizumab OR dostarlimab OR nivolumab OR ipilimumab OR larotrectinib OR entrectinib OR selitrectinib OR repotrectinib OR encorafenib OR binimetinib OR cetuximab OR dabrafenib OR trametinib OR vemurafenib OR "trastuzumab deruxtecan" OR T-DXd OR "trastuzumab emtansine" OR T-DM1 OR "antibody-drug conjugate" )

**-EU Clinical Trials Register (EudraCT/EU CTR)**

- Platform: EU CTR

- Coverage window: All years to 2025-10-21

- Filters/limits: Recruitment status = All; Phase II-III prioritized (no formal phase filter applied); no language limits

- Verbatim query:

(neoplasm OR "solid tumour" OR carcinoma) AND ( "microsatellite instability" OR MSI-H OR dMMR OR "mismatch repair deficiency" OR "tumour mutational burden" OR TMB OR hypermutat OR NTRK1 OR NTRK2 OR NTRK3 OR "TRK fusion" OR "BRAF V600E" OR "BRAF V600K" OR "BRAF V600R" OR "BRAF V600D" OR HER2 OR ERBB2 OR "HER2-low" ) AND ( pembrolizumab OR dostarlimab OR nivolumab OR ipilimumab OR larotrectinib OR entrectinib OR selitrectinib OR repotrectinib OR encorafenib OR binimetinib OR cetuximab OR dabrafenib OR trametinib OR vemurafenib OR "trastuzumab deruxtecan" OR T-DXd OR "trastuzumab emtansine" OR T-DM1 OR "antibody-drug conjugate" )

**- WHO ICTRP (International Clinical Trials Registry Platform)**

- Platform: WHO ICTRP Search Portal

- Coverage window: All years to 2025-10-21

- Filters/limits: Aggregator across registries; no language limits

- Verbatim query:

Condition: neoplasm OR "solid tumor" OR carcinoma

Intervention:

(pembrolizumab OR dostarlimab OR nivolumab OR ipilimumab OR larotrectinib OR entrectinib OR selitrectinib OR repotrectinib OR encorafenib OR binimetinib OR cetuximab OR dabrafenib OR trametinib OR vemurafenib OR "trastuzumab deruxtecan" OR T-DXd OR "trastuzumab emtansine" OR T-DM1 OR "antibody-drug conjugate")

Keywords:

MSI-H OR dMMR OR "microsatellite instability" OR "mismatch repair deficiency" OR "tumor mutational burden" OR TMB OR hypermutat

OR NTRK1 OR NTRK2 OR NTRK3 OR "TRK fusion"

OR "BRAF V600E" OR "BRAF V600K" OR "BRAF V600R" OR "BRAF V600D"

OR HER2 OR ERBB2 OR "HER2-low"

**- ASCO Annual Meeting**

- Platform: ASCO Meeting Library and site-restricted Google queries

- Coverage window: 2015-2025

- Filters/limits: Abstracts; screened for comparative content

- Verbatim site-restricted query (Google):

site:meetinglibrary.asco.org

("pembrolizumab" OR "dostarlimab" OR "nivolumab" OR "ipilimumab" OR "larotrectinib" OR "entrectinib" OR "selitrectinib" OR "repotrectinib" OR "encorafenib" OR "binimetinib" OR "cetuximab" OR "dabrafenib" OR "trametinib" OR "vemurafenib" OR "trastuzumab deruxtecan" OR "T-DXd" OR "trastuzumab emtansine" OR "T-DM1") AND ("MSI" OR "dMMR" OR "microsatellite instability" OR "TMB" OR "hypermutated" OR "NTRK" OR "TRK fusion" OR "BRAF V600" OR "HER2" OR "HER2-low") AND (random OR trial OR phase OR cohort OR comparative OR controlled)

**- ESMO Annual Congress**

- Platform: ESMO websites and site-restricted Google queries

- Coverage window: 2015-2025

- Verbatim site-restricted query (Google):

site:esmo.org OR site:oncologypro.esmo.org

("trastuzumab deruxtecan" OR "T-DXd" OR "pembrolizumab" OR "dostarlimab" OR "nivolumab" OR "larotrectinib" OR "entrectinib" OR "encorafenib" OR "dabrafenib" OR "trametinib") AND ("MSI" OR "dMMR" OR "microsatellite instability" OR "TMB" OR "hypermutated" OR "NTRK" OR "TRK fusion" OR "BRAF V600" OR "HER2" OR "HER2-low") AND (random OR trial OR phase OR cohort OR comparative OR controlled)

**- AACR Annual Meeting**

- Platform: AACR websites and site-restricted Google queries

- Coverage window: 2015-2025

- Verbatim site-restricted query (Google):

site:aacr.org OR site:abstractsonline.com

("pembrolizumab" OR "dostarlimab" OR "nivolumab" OR "ipilimumab" OR "larotrectinib" OR "entrectinib" OR "selitrectinib" OR "repotrectinib" OR "encorafenib" OR "binimetinib" OR "cetuximab" OR "dabrafenib" OR "trametinib" OR "trastuzumab deruxtecan" OR "T-DXd") AND ("MSI" OR "dMMR" OR "microsatellite instability" OR "TMB" OR "hypermutated" OR "NTRK" OR "TRK fusion" OR "BRAF V600" OR "HER2" OR "HER2-low") AND (random OR trial OR cohort OR comparative OR controlled)

**- medRxiv**

- Platform: medRxiv.org search

- Coverage window: Inception to 2025-10-21

- Filters/limits: Preprints; screened for comparative study-level data

- Verbatim query (site search):

("pembrolizumab" OR "dostarlimab" OR "nivolumab" OR "larotrectinib" OR "entrectinib" OR "encorafenib" OR "cetuximab" OR "dabrafenib" OR "trametinib" OR "trastuzumab deruxtecan" OR "T-DXd") AND ("MSI" OR "dMMR" OR "microsatellite instability" OR "TMB" OR "hypermutated" OR "NTRK" OR "TRK fusion" OR "BRAF V600" OR "HER2" OR "HER2-low") AND (random OR trial OR cohort OR comparative OR controlled)

**- bioRxiv**

- Platform: bioRxiv.org search

- Coverage window: Inception to 2025-10-21

- Filters/limits: Preprints; screened for comparative study-level data

- Verbatim query:

("pembrolizumab" OR "dostarlimab" OR "nivolumab" OR "larotrectinib" OR "entrectinib" OR "encorafenib" OR "cetuximab" OR "dabrafenib" OR "trametinib" OR "trastuzumab deruxtecan" OR "T-DXd") AND ("MSI" OR "dMMR" OR "microsatellite instability" OR "TMB" OR "hypermutated" OR "NTRK" OR "TRK fusion" OR "BRAF V600" OR "HER2" OR "HER2-low") AND (random OR trial OR cohort OR comparative OR controlled)

**- ProQuest**

- Platform: ProQuest (multi-database; scholarly works)

- Coverage window: All years to 2025-10-21

- Filters/limits: Keyword and proximity operators; comparative-study terms retained; no language limits

- Verbatim search string

((neoplasm OR tumor OR carcinoma OR "solid tumor") AND ( ("microsatellite NEAR/3 instab" OR MSI-H OR dMMR OR "mismatch NEAR/3 repair NEAR/3 deficien") OR ("tumor NEAR/3 mutational NEAR/3 burden" OR TMB OR hypermutat) OR (NTRK1 OR NTRK2 OR NTRK3 OR "neurotrophic NEAR/3 tyrosine NEAR/3 receptor NEAR/3 kinase" OR "TRK NEAR/2 fusion") OR (BRAF OR "BRAF NEAR/1 V600E" OR "BRAF NEAR/1 V600K" OR "BRAF NEAR/1 V600R" OR "BRAF NEAR/1 V600D") OR (HER2 OR ERBB2 OR "HER2-low" OR "IHC NEAR/1 1+" OR "IHC NEAR/1 2+") ) AND ( pembrolizumab OR dostarlimab OR nivolumab OR ipilimumab OR larotrectinib OR entrectinib OR selitrectinib OR repotrectinib OR encorafenib OR binimetinib OR cetuximab OR dabrafenib OR trametinib OR vemurafenib OR "trastuzumab NEAR/1 deruxtecan" OR T-DXd OR "trastuzumab NEAR/1 emtansine" OR T-DM1 OR "antibody-drug NEAR/1 conjugate" ) AND ( random OR trial OR phase NEAR/2 (II OR III OR 2 OR 3) OR cohort OR "case-control" OR observational OR comparative OR controlled ))

**- Google Scholar**

- Platform: Google Scholar

- Coverage window: All years to 2025-10-21

- Filters/limits: None; first 1000 records screened; date filter applied manually when needed

- Verbatim query:

"solid tumor" OR neoplasm OR carcinoma ("microsatellite instability" OR MSI-H OR dMMR OR "mismatch repair deficiency" OR "tumor mutational burden" OR TMB OR hypermutated OR NTRK OR "TRK fusion" OR "BRAF V600" OR HER2 OR ERBB2 OR "HER2-low") ("pembrolizumab" OR "dostarlimab" OR "nivolumab" OR "ipilimumab" OR "larotrectinib" OR "entrectinib" OR "selitrectinib" OR "repotrectinib" OR "encorafenib" OR "binimetinib" OR "cetuximab" OR "dabrafenib" OR "trametinib" OR "trastuzumab deruxtecan" OR "T-DXd" OR "trastuzumab emtansine" OR "T-DM1") randomized OR randomised OR trial OR cohort OR "case-control" OR comparative

**- CrossRef**

- Platform: CrossRef

- Coverage window: All years to 2025-10-21

- Filters/limits: first 1000 records screened; type=journal-article; no language limits

- Verbatim:

(neoplasm OR "solid tumor" OR carcinoma) AND ("microsatellite instability" OR MSI-H OR dMMR OR "mismatch repair deficiency" OR "tumor mutational burden" OR TMB OR hypermutated OR NTRK OR "TRK fusion" OR "BRAF V600" OR HER2 OR ERBB2 OR "HER2-low") AND (pembrolizumab OR dostarlimab OR nivolumab OR ipilimumab OR larotrectinib OR entrectinib OR selitrectinib OR repotrectinib OR encorafenib OR binimetinib OR cetuximab OR dabrafenib OR trametinib OR vemurafenib OR "trastuzumab deruxtecan" OR T-DXd OR "trastuzumab emtansine" OR T-DM1)

**- Semantic Scholar**

- Platform: Semantic Scholar

- Coverage window: All years to 2025-10-21

- Filters/limits: Keyword-based; no language limits

- Verbatim query:

("solid tumor" OR neoplasm OR carcinoma) AND ("microsatellite instability" OR MSI-H OR dMMR OR "mismatch repair deficiency" OR "tumor mutational burden" OR TMB OR hypermutated OR NTRK OR "TRK fusion" OR "BRAF V600" OR HER2 OR ERBB2 OR "HER2-low") AND ("pembrolizumab" OR "dostarlimab" OR "nivolumab" OR "ipilimumab" OR "larotrectinib" OR "entrectinib" OR "selitrectinib" OR "repotrectinib" OR "encorafenib" OR "binimetinib" OR "cetuximab" OR "dabrafenib" OR "trametinib" OR "trastuzumab deruxtecan" OR "T-DXd" OR "trastuzumab emtansine" OR "T-DM1") AND (randomized OR trial OR cohort OR "case-control" OR comparative)

**- LENS.org (Scholarly Works)**

- Platform: LENS.org (Scholarly Works)

- Coverage window: All years to 2025-10-21

- Filters/limits: Scholarly works; keyword-based search; no language limits

- Verbatim query:

(neoplasm OR "solid tumor" OR carcinoma) AND ("microsatellite instability" OR MSI-H OR dMMR OR "mismatch repair deficiency" OR "tumor mutational burden" OR TMB OR hypermutated OR NTRK OR "TRK fusion" OR "BRAF V600" OR HER2 OR ERBB2 OR "HER2-low") AND (pembrolizumab OR dostarlimab OR nivolumab OR ipilimumab OR larotrectinib OR entrectinib OR selitrectinib OR repotrectinib OR encorafenib OR binimetinib OR cetuximab OR dabrafenib OR trametinib OR vemurafenib OR "trastuzumab deruxtecan" OR T-DXd OR "trastuzumab emtansine" OR T-DM1) AND (randomized OR trial OR cohort OR "case-control" OR comparative OR controlled)

Peer review of the search (PRESS) was completed on June 10, 2025, by a health sciences information specialist. Revisions included the addition of HER2-low terminology and ERBB2 synonyms such as “HER2-low” and “ERBB2,” the inclusion of next-generation TRK inhibitors like selitrectinib and repotrectinib, and expanded TMB-related synonyms like “hypermutated.” Field tags were corrected, examples include PubMed [Mesh], [tiab]; Scopus TITLE-ABS-KEY; and Web of Science TS=. Proximity operators in Web of Science (NEAR/x) and Scopus (W/n) were refined for better precision.

The search strategy emphasized including trial- and observational-comparative terms to increase precision while maintaining recall; single-arm evidence was not excluded. Queries jointly captured biomarkers and agents such as PD-1 inhibitors (pembrolizumab, dostarlimab, nivolumab, ipilimumab) for MSI-H/dMMR and TMB-high, TRK inhibitors (larotrectinib, entrectinib; additionally, selitrectinib and repotrectinib in resistance contexts), BRAF-directed regimens (encorafenib with or without cetuximab and chemotherapy; dabrafenib plus trametinib; vemurafenib), and HER2-targeted antibody-drug conjugates like trastuzumab deruxtecan, including HER2-low.

The date limits were uniformly applied across bibliographic databases. No language restrictions were imposed during searching; translation was performed when feasible. Broad condition terms such as “neoplasm” and “solid tumor” were combined with biomarker and agent keywords in registry searches, encompassing interventional and observational studies across all recruitment statuses. Grey literature sources were accessed via site-restricted queries to ensure retrieval from meeting libraries; these strategies are documented to ensure reproducibility. Deduplication was performed using EndNote and Rayyan, applying exact and fuzzy matching on titles, authors, DOI/registry IDs, years, and sources; near-duplicates were verified manually.

Limitations included challenges with Google Scholar reproducibility, mitigated through documented date stamps and query strings, primarily used for targeted citation checks and screening the first 1000 results. Resource constraints prevented systematic searches of regional databases like CNKI, acknowledging potential language bias in the manuscript. Embase was not systematically searched; however, coverage was partially addressed via Scopus and Web of Science.

**4. Date stamps and update procedures**

Final comprehensive database searches were completed by October 20, 2025, including MEDLINE, CENTRAL, Web of Science, and Scopus. Registries and grey literature sources, including ClinicalTrials.gov, EU CTR, WHO ICTRP, conferences, preprint servers, and grey literature platforms, were searched by October 21, 2025. The analysis lock was also set on October 21, 2025. Weekly monitoring of key biomarker-agent pairs will continue until the analysis lock.

**5. Record yields by source (searches through 2025-10-21)**

The sources and screening process for the collected records from MEDLINE via PubMed yielded 1,839 records from 2000 to the search date, focusing on human studies published since January 1, 2000. Cochrane CENTRAL contributed 1,517 trial records from inception to the search date. The Web of Science Core Collection provided 3,993 records, including SCI-EXPANDED, SSCI, and ESCI documents from 2000 onward. Scopus added 5,903 records, covering Articles, Articles in Press, and Conference Papers from 2000 onward. OpenAlex retrieved 1,545 journal articles published between 2000 and October 20, 2025. ClinicalTrials.gov listed 91 records, including interventional and observational studies, with recruitment and results data, across all age groups. EU CTR supplied 85 records, emphasizing Phase II-III trials with various recruitment statuses. WHO ICTRP presented 42 records from its aggregated search. The ASCO Annual Meeting contributed 385 records from 2015 to 2025, while ESMO and AACR contributed 125 and 726 records, respectively, from the same period. MedRxiv yielded 28 records, and bioRxiv listed 112, both from inception to the search date. ProQuest returned 52,102 records using broad keywords and proximity filters across all years. Google Scholar and CrossRef provided the first 1,000 records each, screened for relevance. Semantic Scholar contributed 2,678 records focused on keyword-based comparative studies, and LENS.org contributed 44,054 records from scholarly works. In total, 117,225 records were identified across all sources. Before screening, 35,249 duplicates were removed, leaving 81,976 records. Titles and abstracts were screened, with 1,511 identified as potentially eligible for full-text review. Four records were not retrieved, and 1,507 full texts were assessed. Exclusions at this stage totaled 1,469, resulting in 38 studies included in the review.

**Supplementary Table S1. Record Yields by Source (Oct 21, 2025)**

| **Source** | **Platform** | **Records Yielded** |
| --- | --- | --- |
| **MEDLINE** | PubMed | 1,839 |
| **CENTRAL** | Cochrane Library | 1,517 |
| **Web of Science** | Core Collection | 3,993 |
| **Scopus** | Elsevier | 5,903 |
| **OpenAlex** | OpenAlex | 1,545 |
| **Grey Literature** | ProQuest / LENS.org | 96,156 |
| **Registries** | CT.gov / EU CTR | 218 |
| **Conferences** | ASCO / ESMO | 1,236 |
| **TOTAL** |  | 117,225 |

ASCO, American Society of Clinical Oncology; CT.gov, ClinicalTrials.gov; ESMO, European Society for Medical Oncology; EU CTR, EU Clinical Trials Register.

**6- Deduplication, consolidation, and audit trail**

The software and workflow employed involved the sequential use of EndNote and Rayyan, followed by manual verification to identify near-duplicates and to perform series-level consolidations. Matching logic included exact matches based on title, first author, DOI or registry identifier, publication year, and source database or platform. Fuzzy matching techniques, such as Levenshtein distance on titles and author-string similarity, were applied to identify near-duplicates, along with considerations of variant DOIs (e.g., early-access versus version-of-record), conference abstracts versus journal articles, and registry records linked to publications.

For multi-source consolidation, registry-to-publication mappings used NCT and EudraCT identifiers linked to journal articles. Preference was given to the most complete, peer-reviewed report for each outcome and time point, with interim analyses retained only when final reports did not supersede them. In cases of multiple reports and overlapping cohorts, the largest and least biased dataset was chosen, including multiple reports from a common registry that spanned distinct time periods or sites. Harmonization of ahead-of-print versions and version-of-record content ensured the latter replaced early-access data, with citation corrections and errata flagged accordingly.

Regarding counts reconciliation and documentation, deduplication reduced the initial 117,225 records to 81,976 unique items before screening. PRISMA flow counts and source-specific yields were documented contemporaneously in search spreadsheets.

Quality control measures included random audits of deduplication sets to verify the removal of true duplicates and ensure near-duplicates were retained only when genuinely distinct. Trial registry entries were cross-verified with publication metadata to prevent double-counting across different sources.

**7- Deviations and ‎ limitations**

There were no significant deviations from the original protocol. Minor changes were made before screening, such as adding HER2-low/ERBB2 terminology and including OpenAlex to enhance citation discovery; these adjustments are recorded in the protocol amendment log. Limitations include that Google Scholar retrieval is not fully reproducible, as it was used for targeted forward citation checks with recorded query strings and timestamps. Also, comprehensive searches of regional databases like CNKI were not conducted due to resource constraints, which may slightly increase the risk of language bias.

**8- Good practice and alignment with reporting standards**

The search and documentation processes strictly adhered to PRISMA 2020 and PRISMA-S guidelines, ensuring they were tailored to the specific clinical question to retrieve comparative studies across different histologies effectively. The development of the search strategy followed established standards set by Cochrane and MECIR, including searches of registries and grey literature to reduce publication and reporting bias. Weekly alerts were maintained continuously until the analysis was finalized.

**‎**

**Supplementary Table S2. Full-Text Exclusions**

| **Exclusion Category** | **Number of Reports** | **Primary Reason** |
| --- | --- | --- |
| **Grey Literature / No Outcomes** | 194 | Protocols, news, no data |
| **Non-Comparative** | 230 | Single-arm, case series |
| **Non-Clinical** | 286 | Preclinical, *in vitro* |
| **Secondary Research** | 86 | Reviews without new data |
| **Ineligible Population** | 178 | Wrong tumor type, pediatric only |
| **Ineligible Intervention** | 164 | Non-biomarker guided |
| **Diagnostic Only** | 37 | Assay validation only |
| **Composite / Unspecified** | 201 | Mixed ineligible reasons |
| **Not Retrieved** | 4 | Full text unavailable |
| **TOTAL EXCLUDED** | 1,469 |  |

**Supplementary Methods: ‎Risk of Bias**

**1. Tools and approach**

We evaluated the risk of bias at the outcome level for all effect estimates incorporated into the quantitative synthesis, including confirmed objective response within 24 weeks, progression-free survival, overall survival, duration of response, grade 3-4 adverse events, and discontinuation due to toxicity. These evaluations were linked to the specific estimand used, such as blinded, independent, central review versus investigator assessment, or intention-to-treat versus per-protocol analyses.

For bias assessment, we used RoB 2 for randomized trials and ROBINS-I for nonrandomized comparative studies, including retrospective cohorts, external controls, and matching-adjusted indirect comparisons. The assessment process involved two independent reviewers who performed duplicate evaluations after calibration. Disagreements were resolved through consensus, with senior adjudication when necessary. We utilized protocols, statistical analysis plans, trial registries, and regulatory summaries to verify prespecified outcomes and identify any selective reporting.

For analytic integration, studies identified as high- or serious-risk of bias were excluded from prespecified sensitivity analyses. Outcomes classified as having a "critical” ROBINS-I risk were not pooled. Furthermore, risk-of-bias categories were examined as moderators in sensitivity analyses, meta-regression analyses, and GRADE judgments.

**2. Summary of domain-level patterns**

Randomized trials generally exhibited low risk across various domains. Randomization and allocation procedures were appropriate, with progression-free survival and objective response rate often adjudicated by blinded independent central review. Open-label designs were mitigated by centralized efficacy review. In cases where investigator-assessed PFS was the primary endpoint, such as in DESTINY‑Breast04, we noted some concerns regarding outcome measurement, but this did not affect the overall direction or clinical conclusions. The crossover in KEYNOTE‑177 reduced the observed overall survival differences without biasing the intention-to-treat analysis; we identified some concerns only regarding the interpretability of the OS result, not the overall findings.

Nonrandomized studies typically carry a moderate-to-serious risk of bias, mainly due to residual confounding factors, such as incomplete adjustment for ECOG performance status, disease burden, prior therapies, and molecular markers like PD‑L1, TMB, or co-mutations. Selection bias was also a concern, related to time-zero alignment, immortal time bias, and heterogeneity across trials in matching-adjusted indirect comparisons. While outcome measurement was generally objective and acceptable, the classification of exposures was adequate within cohorts but varied across external controls. These results provided context and insights into effect modification but did not primarily drive pooled estimates.

**3. Per-study, per‑domain risk-of-bias matrix (concise justifications)**

**Supplementary Table S3. Randomized studies (RoB 2) and Nonrandomized studies (ROBINS-I) per-study, per-domain risk-of-bias matrix**

| **Randomized studies (RoB 2)** | | | | | | | | | | | | | | | | | |
| --- | --- | --- | --- | --- | --- | --- | --- | --- | --- | --- | --- | --- | --- | --- | --- | --- | --- |
| **Study** | | **Outcome focus** | | **Randomization** | | | **Deviations** | **Missing data** | | **Outcome measurement** | | | **Selection of reported results** | | **Overall** | | |
| **KEYNOTE-177 (André 2020/2025)** | | PFS/OS/ORR | | Low | | | Low | Low | | Low | | | Some concerns (OS) | | Low (PFS/ORR); Low-some concerns (OS) | | |
| **CheckMate 8HW (André 2024)** | | PFS (RMST)/Safety | | Low | | | Low | Low | | Low | | | Low | | Low | | |
| **RUBY overall (Mirza 2023; Powell 2024)** | | PFS/OS | | Low | | | Low | Low | | Low | | | Low | | Low | | |
| **RUBY dMMR subset (Powell 2025)** | | PFS/OS/ORR/DoR | | Low | | | Low | Low | | Low | | | Low | | Low | | |
| **DESTINY-Breast04 (Modi 2025; Narayan 2023; Bardia 2024 HR+)** | | PFS/OS/ORR/Safety | | Low | | | Low | Low | | Some concerns | | | Low | | Low | | |
| **DESTINY-Breast02 (André 2023)** | | PFS/OS/ORR/Safety | | Low | | | Low | Low | | Low | | | Low | | Low | | |
| **DESTINY-Breast03 Asian subgroup (Iwata 2024)** | | PFS/OS/ORR/Safety | | Low | | | Low | Low | | Low | | | Low | | Low | | |
| **DESTINY-Gastric01 (Shitara 2020)** | | ORR/PFS/OS/Safety | | Low | | | Low | Low | | Low | | | Low | | Low | | |
| **DESTINY-Gastric04 (Shitara 2025)** | | ORR/PFS/OS/Safety | | Low | | | Low | Low | | Low | | | Low | | Low | | |
| **BEACON CRC (Kopetz 2019)** | | OS/PFS/ORR/Safety | | Low | | | Low | Low | | Low | | | Low | | Low | | |
| **BREAKWATER (Elez 2025)** | | OS/PFS/ORR/Safety | | Low | | | Low | Low | | Low | | | Low | | Low | | |
| **SWOG S1406 (Kopetz 2021)** | | PFS/ORR/Safety | | Low | | | Low | Low | | Low | | | Low | | Low | | |
| **EMILIA (Verma 2012)** | | OS/PFS/ORR/Safety | | Low | | | Low | Low | | Low | | | Low | | Low | | |
| **Nonrandomized studies (ROBINS-I)** | | | | | | | | | | | | | | | | | |
| **Study** | **Outcome focus** | | **Confounding** | | **Selection** | **Classification** | | | **Deviations** | | **Missing** | **Outcome measurement** | | **Selection of reported results** | | **Overall** |  |
| **FRONT-BRAF NSCLC (Di Federico 2025)** | OS/PFS/ORR/Safety | | Serious | | Moderate | Low | | | Low | | Moderate | Low | | Some concerns | | Serious |  |
| **GARNET vs EHR control (Goulden 2023)** | OS/TTD | | Serious | | Moderate | Moderate | | | Low | | Moderate | Moderate | | Some concerns | | Serious |  |
| **MAIC larotrectinib vs entrectinib (García-Foncillas 2022)** | OS/PFS/ORR/DoR | | Serious | | Moderate | Moderate | | | Low | | Moderate | Moderate | | Some concerns | | Serious |  |
| **Schettini 2021 (Italian HER2+ mBC)** | PFS/OS | | Serious | | Moderate | Low | | | Low | | Moderate | Moderate | | Some concerns | | Serious |  |
| **Chen J 2025 (MSI-H/dMMR mCRC)** | ORR/PFS/OS | | Serious | | Moderate | Low | | | Low | | Moderate | Moderate | | Some concerns | | Serious |  |
| **Hamidi 2024 (ATC DT vs DTP)** | OS/PFS/ORR/Safety | | Serious | | Moderate | Low | | | Low | | Moderate | Moderate | | Some concerns | | Serious |  |
| **Hill 2023 (Endometrial real-world)** | TTNT/OS | | Serious | | Moderate | Low | | | Low | | Moderate | Moderate | | Some concerns | | Serious |  |
| **André 2024 pooled T-DXd brain mets** | iORR/CNS-PFS/Safety | | Moderate | | Moderate | Low | | | Low | | Low | Low | | Low | | Moderate |  |

RoB 2, Cochrane Risk of Bias 2 tool for randomized trials; ROBINS-I, Risk Of Bias In Non-randomized Studies of Interventions; PFS, Progression-Free Survival; OS, Overall Survival; ORR, Objective Response Rate; RMST, Restricted Mean Survival Time; BICR, Blinded Independent Central Review; MMR, Mismatch Repair; dMMR, Deficient Mismatch Repair; SAP, Statistical Analysis Plan; DoR, Duration of Response; ILD, Interstitial Lung Disease; ADCs, Antibody-Drug Conjugates; HER2, Human Epidermal Growth Factor Receptor 2; HER2+, Human Epidermal Growth Factor Receptor 2-Positive; HR+, Hormone Receptor-Positive; CNS, Central Nervous System; AEs, Adverse Events; RCT, Randomized Controlled Trial; NSCLC, Non-Small Cell Lung Cancer; EHR, Electronic Health Record; IPTW, Inverse Probability of Treatment Weighting; PSM, Propensity Score Matching; MAIC, Matched Adjusted Indirect Comparison; TTD, Time to Treatment Discontinuation; TTNT, Time to Next Treatment; TMB, Tumor Mutational Burden; ATC, Anaplastic Thyroid Carcinoma; DT, Dabrafenib plus Trametinib; DTP, Dabrafenib, Trametinib plus Pembrolizumab; NLR, Neutrophil-to-Lymphocyte Ratio; mBC, Metastatic Breast Cancer; mCRC, Metastatic Colorectal Cancer; ICI, Immune Checkpoint Inhibitor; iORR, Intracranial Objective Response Rate; CNS-PFS, Central Nervous System Progression-Free Survival; PD-L1, Programmed Death-Ligand 1; BRAF, B-Raf Proto-Oncogene Serine/Threonine Kinase; TP53, Tumor Protein p53; mFOLFOX6, Modified FOLFOX6 (5-Fluorouracil, Leucovorin, Oxaliplatin) Regimen; T-DXd, Trastuzumab Deruxtecan; CRC, Colorectal Cancer.

**4. Extended rationale and cross-checks**

For each study and primary outcome, we cite the exact protocol or registry references, such as NCT02563002 [KEYNOTE‑177], NCT03981796 [RUBY], NCT03523585 [DESTINY‑Breast02], NCT03329690 [DESTINY‑Gastric01], NCT04704934 [DESTINY‑Gastric04], NCT02928224 [BEACON], and NCT04607421 [BREAKWATER]. We also include details on adjudication processes, covering BICR charters and RMST specifications, crossing over policies, and controls for multiplicity. Nonrandomized studies are described using covariate lists, propensity and balance diagnostics, and features of target-trial emulation, such as time-zero alignment and contemporaneity, along with discussions of residual confounding and the risk of immortal time bias. For ADC programs, adjudication procedures for ILD/pneumonitis are summarized, with regional variations explained in context.

**5. Influence on synthesis and certainty**

Excluding high- and serious-risk studies resulted in narrower confidence intervals and reduced heterogeneity; however, it did not alter the overall conclusions. The primary randomized evidence, supported by BICR adjudication, served as the basis for pooled efficacy assessments, as seen in studies such as KEYNOTE-177, CheckMate 8HW, RUBY, DESTINY, BEACON, and BREAKWATER. There is a potential for bias in these findings. Investigator-assessed endpoints might slightly favor active treatment arms for subjective measures. Additionally, crossover in KEYNOTE-177 probably biases overall survival results toward no difference. Observational confounding factors can also artificially inflate perceived benefits. Therefore, the GRADE assessment was downgraded: for OS in cases of crossover (moderate certainty) and for indirect or observational comparisons (low to moderate certainty).

**6. Reporting bias assessment (small-study effects/publication ‎bias) ‎**

Small-study effects were assessed using contour-enhanced ‎funnel plots and regression-based asymmetry tests, applied only ‎when the number of contributing effect sizes met a prespecified ‎minimum (k ≥ 10). For dichotomous outcomes synthesized as risk ‎ratios (RR), analyses were conducted on the log risk ratio (log RR) ‎scale with corresponding standard errors; for time-to-event ‎outcomes synthesized as hazard ratios (HR), analyses were ‎conducted on the log hazard ratio (log HR) scale with ‎corresponding standard errors. Egger’s regression test was used as the primary asymmetry test (R: metafor package; regtest function), with the Harbord and Peters tests used as sensitivity checks for binary outcomes where appropriate. To further assess the robustness of findings against potential publication bias, selection model approaches (specifically the Vevea & Hedges weight-function model) were applied as exploratory sensitivity analyses for eligible syntheses exhibiting visual or statistical asymmetry. For pooled analyses with k < 10, formal asymmetry testing was not performed; funnel plots were instead interpreted qualitatively with explicit caution regarding low statistical power and reliability. Full specifications, including test type, metric, software, eligibility criteria, and selection model results, are detailed in Supplementary Table S8.‎

**Supplementary Table S4. Trial-Report Linkage and Endpoint Contribution Map (to prevent double-counting across multiple publications/updates)**

| **Underlying Trial / Program (NCT ID)** | **Linked Reports Captured** | **Endpoints Contributed to Synthesis** | **Selection Rule & Double-Counting Prevention Note** |
| --- | --- | --- | --- |
| **KEYNOTE-177 (NCT02563002)** | 1. André T et al., 2020 NEJM (Primary)  2. André T et al., 2025 Ann Oncol (5-yr update)  3. Casak SJ et al., 2021 Clin Cancer Res (FDA)  4. Yoshino T et al., 2023 Cancer Sci (Asian) | PFS, ORR, Safety: André 2020  OS, DoR: André 2025  Subgroup (Context): Yoshino 2023 | Rule: Most mature peer-reviewed report used per endpoint.  Note: André 2020 used for PFS/ORR (primary estimand); André 2025 used for final OS. FDA summary (Casak) used for triangulation only. Yoshino 2023 analyzed as a separate regional subgroup, not pooled with the global ITT set. |
| **CheckMate 8HW (NCT04008030)** | André T et al., 2024 NEJM | PFS (RMST), Safety | Rule: Primary interim analysis (BICR).  Note: Single report used. RMST reconstruction performed due to non-proportional hazards. |
| **DESTINY-Breast04 (NCT03734029)** | 1. Modi S et al., 2025 Nat Med (Long-term)  2. Narayan P et al., 2023 JCO (FDA)  3. Yamashita T et al., 2024 Breast Cancer (Asian) | OS, PFS, ORR, Safety: Modi 2025 | Rule: Most mature long-term data prioritized.  Note: Modi 2025 is the primary source for global pooling. Asian subgroup (Yamashita) and FDA summary (Narayan) used for sensitivity/context only. |
| **DESTINY-Breast02 (NCT03523585)** | André F et al., 2023 Lancet | PFS, OS, ORR, Safety | Rule: Primary RCT report.  Note: Single contribution to HER2+ mBC post-T-DM1 synthesis. |
| **DESTINY-Gastric01 (NCT03329690)** | Shitara K et al., 2020 NEJM | ORR, PFS, OS, Safety | Rule: Primary RCT report.  Note: Single contribution. |
| **DESTINY-Gastric04 (NCT04704934)** | Shitara K et al., 2025 NEJM | OS, PFS, ORR, Safety | Rule: Primary RCT report.  Note: Single contribution. |
| **RUBY (NCT03981796)** | 1. Mirza MR et al., 2023 NEJM (Primary)  2. Powell MA et al., 2024 Ann Oncol (OS update)  3. Powell MA et al., 2025 Gynecol Oncol (dMMR subset) | PFS, Safety: Mirza 2023  OS: Powell 2024 | Rule: Most mature specific report per endpoint.  Note: Mirza 2023 used for PFS/Safety; Powell 2024 for final OS. Powell 2025 used only for dMMR-specific stratification logic, not duplicated in overall analysis. |
| **BEACON CRC (NCT02928224)** | 1. Kopetz S et al., 2019 NEJM  2. Stintzing S et al., 2022 Clin Colorectal Cancer | OS, PFS, ORR: Kopetz 2019  Context: Stintzing 2022 | Rule: Primary RCT for efficacy.  Note: Kopetz 2019 used for primary pooled efficacy to ensure intention-to-treat sample consistency. Stintzing 2022 used for QoL context only. |
| **DESTINY Pooled Brain Mets** | André F et al., 2024 Ann Oncol | Intracranial ORR, CNS-PFS | Rule: Pooled subset analysis.  Note: Used only for CNS-specific endpoints not available in the main trial reports. Not pooled with individual trials for systemic endpoints. |
| **T-DXd Post-Endocrine** | Bardia A et al., 2024 NEJM | PFS, ORR, Safety | Rule: Primary RCT report.  Note: Distinct trial from DESTINY-Breast04 (different population); analyzed as a separate entry. |
| **SWOG S1406 (NCT02164916)** | Kopetz S et al., 2021 JCO | PFS, ORR, Safety | Rule: Primary RCT report.  Note: Single contribution. Crossover noted for OS interpretation. |
| **BREAKWATER (NCT04607421)** | Elez E et al., 2025 NEJM | PFS, OS, ORR, Safety | Rule: Primary RCT report.  Note: Single contribution. |
| **EMILIA (NCT00829166)** | Verma S et al., 2012 NEJM | PFS, OS, ORR, Safety | Rule: Primary RCT report.  Note: Single contribution. |
| **TH3RESA (NCT01419197)** | Wildiers H et al., 2013 Eur J Cancer (Abstract) | PFS, ORR | Rule: Conference abstract.  Note: Used as supportive evidence; acknowledged as interim/less detailed. |
| **GARNET (NCT02715284)** | Goulden S et al., 2023 JHEOR (vs External Control)  2. Mathews C et al., 2022 Oncologist (Indirect) | OS, TTD: Goulden 2023  Context: Mathews 2022 | Rule: External control analysis prioritized.  Note: Goulden 2023 used for OS/TTD hazard ratios. Mathews 2022 used for indirect context only. |

BICR, Blinded Independent Central Review; ITT, Intention-to-Treat; RMST, Restricted Mean Survival Time; OS, Overall Survival; PFS, Progression-Free Survival; ORR, Objective Response Rate; DoR, Duration of Response; QoL, Quality of Life; CNS, Central Nervous System; TTD, Time to Treatment Discontinuation.

**Supplementary Table S5. IPD from KM Reconstructions Transparency**

| **Study ID** | **Outcome** | **Reason** | **Software** | **Validation Metric** | **Use** |
| --- | --- | --- | --- | --- | --- |
| **CheckMate 8HW** | PFS (RMST) | Non-PH | IPD from KM ‎ | < 2% deviation | Primary |
| **KEYNOTE-177** | PFS (RMST) | Crossing curves | IPD from KM ‎ | < 1% deviation | Complementary |
| **DESTINY-Breast04** | PFS (RMST) | Late separation | IPD from KM | < 3% deviation | Complementary |
| **Tabernero 2016** | PFS (HR) | No HR reported | Web Plot Digitizer | Matched survival | Sensitivity |
| **Schettini 2021** | OS (HR) | Observational | IPD from KM | < 5% deviation | Sensitivity |

HR, Hazard Ratio; IPD, Individual Patient Data; KM, Kaplan-Meier; PFS, Progression-Free Survival; PH, Proportional Hazards; RMST, Restricted Mean Survival Time.

**Supplementary Table S6. Covariate Balance Diagnostics (Standardized Mean Differences) for External Control Studies**

| **Covariate** | **Before Weighting (Unadjusted)** | | | **After Weighting (IPTW)** | | |
| --- | --- | --- | --- | --- | --- | --- |
|  | Trial Mean | Control Mean | SMD | Trial Mean | Control Mean | SMD |
| **Age (mean years)** | 63.5 | 67.1 | 0.32 | 64.2 | 64.0 | 0.02 |
| **ECOG PS 0 (%)** | 56.0% | 34.0% | 0.45 | 45.0% | 46.2% | 0.03 |
| **Histology: Endometrioid (%)** | 72.0% | 58.0% | 0.30 | 65.0% | 64.5% | 0.01 |
| **Histology: Serous/Clear Cell (%)** | 8.0% | 15.0% | 0.22 | 11.0% | 11.2% | 0.01 |
| **FIGO Stage IV (%)** | 61.0% | 48.0% | 0.26 | 54.5% | 55.0% | 0.01 |
| **Prior Lines of Therapy (Median)** | 1.0 | 1.0 | 0.05 | 1.0 | 1.0 | <0.01 |
| **Race: White (%)** | 82.0% | 70.0% | 0.28 | 76.0% | 75.5% | 0.01 |

ECOG PS, Eastern Cooperative Oncology Group Performance Status; FIGO, International Federation of Gynecology and Obstetrics; IPTW, Inverse Probability of Treatment Weighting; SMD, Standardized Mean Difference. Note: SMD > 0.10 indicates meaningful imbalance; SMD < 0.10 indicates negligible imbalance. ‎Study: Goulden S et al., 2023 (GARNET Trial vs. Real-World External Control), Population: dMMR/MSI-H Endometrial Cancer (Post-Platinum).‎

**Supplementary Table S7. Baseline Characteristics of Included Studies**

| **Study ID** | **Median Age (yr)** | **ECOG 0/1 (%)** | **Prior Lines** | **Visceral Mets (%)** | **Brain Mets (%)** | **Biomarker Method** |
| --- | --- | --- | --- | --- | --- | --- |
| **KEYNOTE-177** | 63 | 49 / 55 | 0 (1L) | NR | Excluded | PCR / IHC (Local) |
| **CheckMate 8HW** | 61 | 58 / 42 | 0 (1L) | NR | Excluded | Central Confirmation |
| **DESTINY-Breast04** | 57 | 56 / 44 | 1-2 | 70 | Excluded ‎† | IHC 1+/2+ ISH- (Central) |
| **DESTINY-Breast02** | 54 | 56 / 43 | 2 | 78 | 18 | IHC 3+ (Central) |
| **RUBY** | 64 | 54 / 46 | 0 (1L) | NR | Excluded | IHC (Local) |
| **BEACON CRC** | 61 | 51 / 45 | 1-2 | 66 | Excluded | PCR (Local/Central) |
| **Shitara 2020** | 66 | 45 / 55 | ≥2 | 90 | Excluded | IHC 3+ / 2+ ISH+ (Central) |
| **Shitara 2025** | 62 | 53 / 47 | 1 | 85 | Excluded | IHC (Central Rebiopsy) |

ECOG, Eastern Cooperative Oncology Group; IHC, Immunohistochemistry; PCR, Polymerase Chain Reaction; 1L, First Line; NR, Not Reported; ‎†, Active brain mets excluded; stable treated allowed. ‎

**Supplementary Table S8. Multiplicity-Adjusted Interaction Results**

| **Moderator** | **Comparison** | **k** | **Unadjusted p** | **Adjusted q (FDR)** | **Interpretation** |
| --- | --- | --- | --- | --- | --- |
| **MMR Status** | dMMR vs pMMR | 2 | < 0.001 | < 0.001 | Robust |
| **ADC Dose** | 5.4 vs 6.4 mg/kg | 4 | 0.041 | 0.082 | Exploratory Trend |
| **Line of Therapy** | 1L vs Later | 2 | 0.032 | 0.075 | Exploratory Trend |
| **Geography** | Japan vs Others | 3 | 0.045 | 0.082 | Exploratory Trend |
| **TMB Assay** | Tissue vs Blood | 2 | 0.070 | 0.112 | Not Significant |

FDR, False Discovery Rate; MMR, Mismatch Repair; TMB, Tumor Mutational Burden.

**Supplementary Table S9. Exact Test Specifications and Synthesis-Level Results for Funnel Plot-Based Assessments**

| **Outcome Family** | **Eligibility (k≥10)** | **Metric** | **Test** | **k** | **p-value** | **Interpretation & Selection Model Check** |
| --- | --- | --- | --- | --- | --- | --- |
| **Confirmed ORR** | Yes | log RR | Egger | 24 | 0.62 | No evidence of bias. |
| **PFS** | Yes | log HR | Egger | 28 | 0.64 | No evidence of bias. |
| **Grade ≥3 AEs** | Yes | log RR | Egger | 21 | 0.40 | No evidence of bias. |
| **Binary Harm (Exploratory)** | Yes | log RR | Egger | 12 | 0.046 | Mild asymmetry.  Sensitivity: Trim-and-fill imputed 2 studies (effect unchanged).  Selection Model (Vevea-Hedges): Adjusted RR 0.88 (95% CI 0.64-1.12) vs. unadjusted RR 0.86; difference negligible.  Robust to selection bias. |

AE, Adverse Event; HR, Hazard Ratio; k, Number of Studies; ORR, Objective Response Rate; PFS, Progression-Free Survival; RR, Risk Ratio. Note: Funnel asymmetry tests are synthesis-level procedures and yield one p-value per synthesis/outcome family. For all other pooled comparisons with k < 10 (including rare harms such as ILD/pneumonitis), formal funnel-based asymmetry testing was not performed, and any funnel visualizations were interpreted qualitatively with explicit caution due to low power and unreliability at small k.

**Supplementary Table S10. Sensitivity Analyses**

| **Synthesis** | **Method Change** | **Pooled Effect (Original)** | **Pooled Effect (Sensitivity)** | **Conclusion** |
| --- | --- | --- | --- | --- |
| **MSI-H PFS** | Fixed Effect | HR 0.60 | HR 0.59 | Robust |
| **HER2-low OS** | Leave-One-Out | HR 0.69 | HR 0.69-0.72 | Robust |
| **BRAF OS** | Exclude Observational | HR 0.53 | HR 0.52 | Robust |
| **Endometrial PFS** | Adjusted Adjudication | HR 0.29 | HR 0.30 | Robust |

HR, Hazard Ratio; MSI-H, Microsatellite Instability-High; OS, Overall Survival; PFS, Progression-Free Survival.

**Supplementary Table S11. Sensitivity Analysis Restricted to Tumor-Agnostic Indications**

| **Metric** | **Primary Analysis (All Included)** | **Restricted Sensitivity (Agnostic Only‎‡‎)** | **Interpretation** |
| --- | --- | --- | --- |
| **Included Studies (k)** | 38 | 14 | Excludes HER2/BRAF histology-anchored |
| **Pooled ORR, RR (CI)** | 2.15 (1.85-2.50) | 1.88 (1.45-2.42) | Robust, slightly lower |
| **Pooled PFS, HR (CI)** | 0.54 (0.48-0.61) | 0.51 (0.40-0.65) | Consistent / Stronger |
| **Pooled OS, HR (CI)** | 0.62 (0.55-0.70) | 0.65 (0.52-0.80) | Consistent |
| **Heterogeneity (I²)** | 68% | 45% | Improved in an agnostic set |

HR, Hazard Ratio; ORR, Objective Response Rate; OS, Overall Survival; PFS, Progression-Free Survival; RR, Risk Ratio. ‎‡ Includes MSI-H, TMB-High, NTRK. Excludes HER2, BRAF.
